# The Levantine Megalithic Building Techniques: A Groundbreaking Method Applied to Menjez’s Monuments (Akkar, Lebanon) from the 4th–3rd Millennium BCE

**DOI:** 10.1007/s10816-024-09654-9

**Published:** 2024-06-03

**Authors:** Méryl Defours Rivoira, Florian Cousseau, Tara Steimer-Herbet

**Affiliations:** Laboratory of Prehistoric Archaeology and Anthropology, Department F.-A. Forel for Environmental and Aquatic Sciences, University of Geneva, Geneva, Switzerland

**Keywords:** Lebanon, Megaliths, Architecture, Buildings archaeology, Geoarchaeology, Basaltic quarry

## Abstract

The aim of this paper is to present the methodology used to study the megalithic architecture of Menjez’s monuments (Akkar, Lebanon), as part of the MEG-A Project - “First megalith builders in the northern Levant” (2022–2025). Twenty-four monuments have been investigated since 2018. The primary objective is to pioneer a comprehensive understanding of the unique Levantine megalithic building techniques and re-establish the “chaînes opératoires,” by determining the builders’ sequence of actions. This groundbreaking methodology originally developed for Western European megalithic monuments, notably in Brittany, France, has been innovatively applied and customized to suit the Levantine context, specifically focusing on the distinctive basaltic monuments of Menjez and its surrounding areas. By using photogrammetry as a tool, the researchers are able to de-construct the megalithic architecture by analyzing the different components of these monuments. Moreover, it is then possible to describe monoliths according to their place within the monument and their geological and geomorphological features. Our work has led us to consider the symbolic aspect expressed in the megalithic architecture of Menjez. Employing this groundbreaking methodology not only yields concrete answers regarding the typology of these monuments but also dramatically reshapes our perception of their construction. It establishes a precise relative chronology for the various architectural phases and, most significantly, reveals the hidden details of the raw material supply chain.

## Introduction

Data on Levantine megalithic architecture has grown considerably since the early 2000s, thanks to a large number of surveys (Armendáriz *et al*., 2008, 2012; Bradbury & Philip, 2011; Fraser *et al*., 2009; Ibáñez *et al*., 2010; Ji, 1997; Khouri *et al*., 2006; Steimer-Herbet & Zuobee, 2014; Steimer-Herbet & Criaud, 2008; Thuesen, 2004). Several thousand megalithic monuments have been documented in the area; however, only 300 of them have been excavated. Digs have been carried out in Israel (Aveni & Mizrachi, 1998; Bahat, 1972; Epstein, 1985; Freikman & Porat, 2017; Vinitzky, 1992), Jordan (Fernandez-Tresguerres Velasco & Junceda Quintana, 1991; Fernandez-Tresguerres Velasco, 2011; Kerner, 2019; Kerner *et al.*, 2017; Polcaro *et al*., 2014; Polcaro & Muniz, 2018; Kołodziejczyk, personal communication), Syria (Braemer *et al*., 2004, 2011; Steimer-Herbet, 2006; Steimer-Herbet & Besse, 2017), and Lebanon (Steimer-Herbet *et al*., 2022; Tallon, 1964). Some researchers have also undertaken a review of the constantly evolving research on this subject (Avner, 2002; Fraser, 2018; Steimer-Herbet, 2004, 2004–2005).

In Menjez, located in the ‘Akkar region of northern Lebanon, we have inaugurated a new Levantine megalithic building techniques methodology, marking a first in the region. This unique methodological approach rests on two fundamental pillars. It relies on the one hand on principles developed in buildings archaeology, as used for the in-depth study of the historical buildings’ architecture, and on the other hand, it involves observing the geological and geomorphological characteristics of monoliths that make up the monuments studied. This methodology is inspired by works carried out in Western Europe, where it has already proved its worth in the analysis of megalithic monuments, providing a new reading of these architectures in volume and 3D. The aim was to adapt it to the specific geology of Menjez, while exploring its potential application in the architectural analysis of megaliths in the ‘Akkar region. This manner to conceiving the study of megalithic monuments represents a total innovation in the Levantine context.

The various studies carried out on Levantine megaliths aim to enhance our understanding of the subject and, in particular, the functioning of these megalithic societies. By proposing this approach to the study of these exceptional architectures, we contribute to improve our knowledge on the gestures of megalith builders and grasp the technological and cultural dynamics that characterized these Levantine societies in the 4th and 3rd millennia BCE.

This article presents the new method, Levantine megalithic building techniques, used to examine in detail the Menjez’s megalithic structures. This approach is illustrated through the analysis of two concrete examples.

## Corpus

Few megalithic sites have been recorded in Lebanese territory, the only known examples being located in the north near Mount Hermel (Tallon, 1959) and in the ‘Akkar (Mouterde, 1940; Tallon, 1958, 1964; Matsumoto & Wada, 2001; Wygnańska, 2018; Steimer, 2000; Steimer-Herbet *et al*., 2018, 2019a, b, 2020). One might expect to find megalithic monuments in southern Lebanon, since the nearby Jaulan Plateau is covered with them, but this is not the case.

**Fig. 1:**
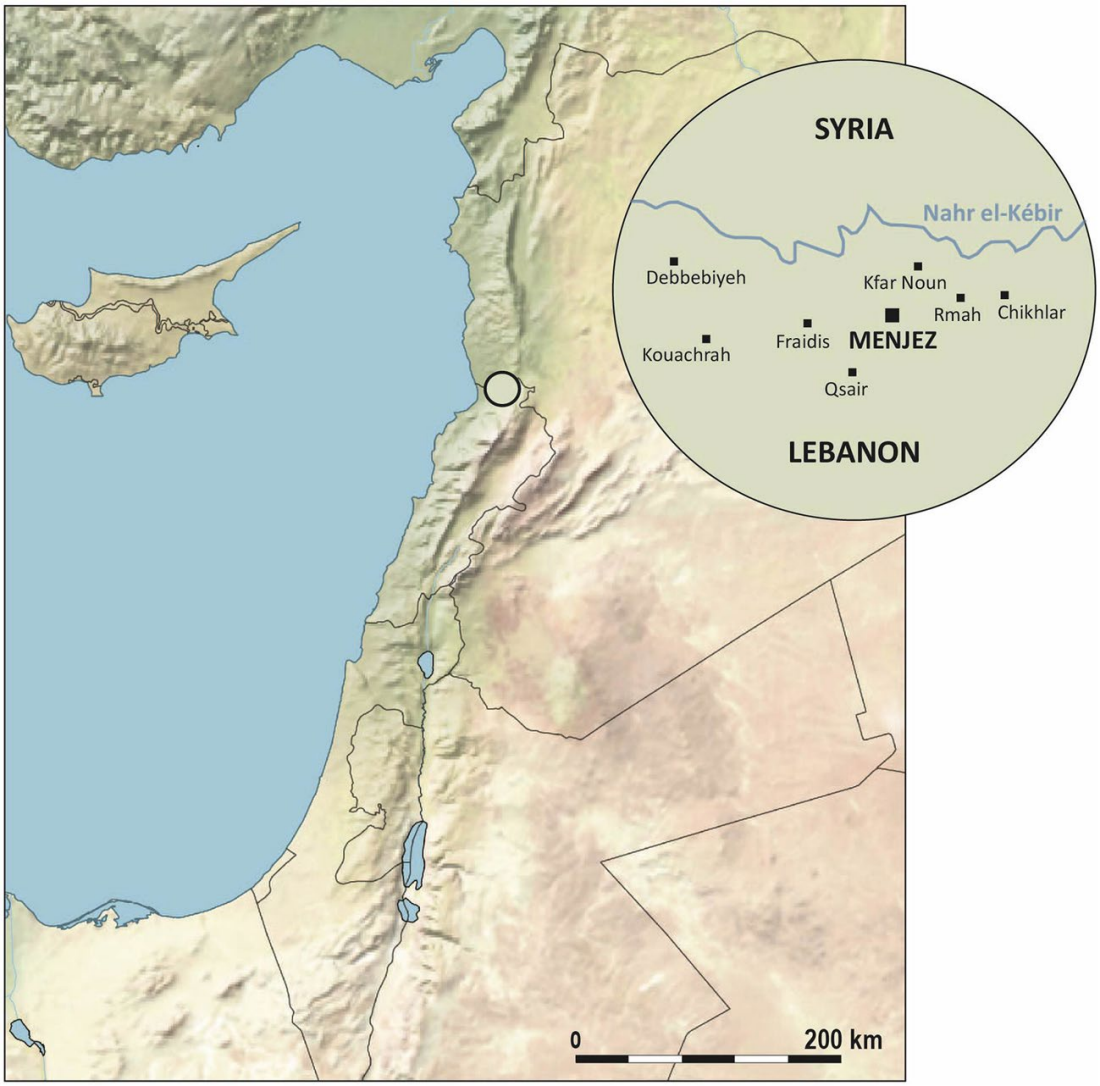
MEG-A Project study area

The modern village of Menjez is located in the ‘Akkar hills, some 3 km south of the Nahr el-Kebir, the river forming the border with Syria (Fig. 1). This area hosts the largest concentration of megalithic monuments in Lebanon (Artin, 2014–2015, p.23), with 87 megalithic tombs documented by M. Tallon in the 1960s (Tallon, 1964). Today, only 45 of these monuments are still visible, alongside 5 houses, structures whose function remains to be determined, and areas of pottery and lithic material concentration (Steimer-Herbet *et al*., 2022; Wygnańska *et al*., 2022).

**Fig. 2:**
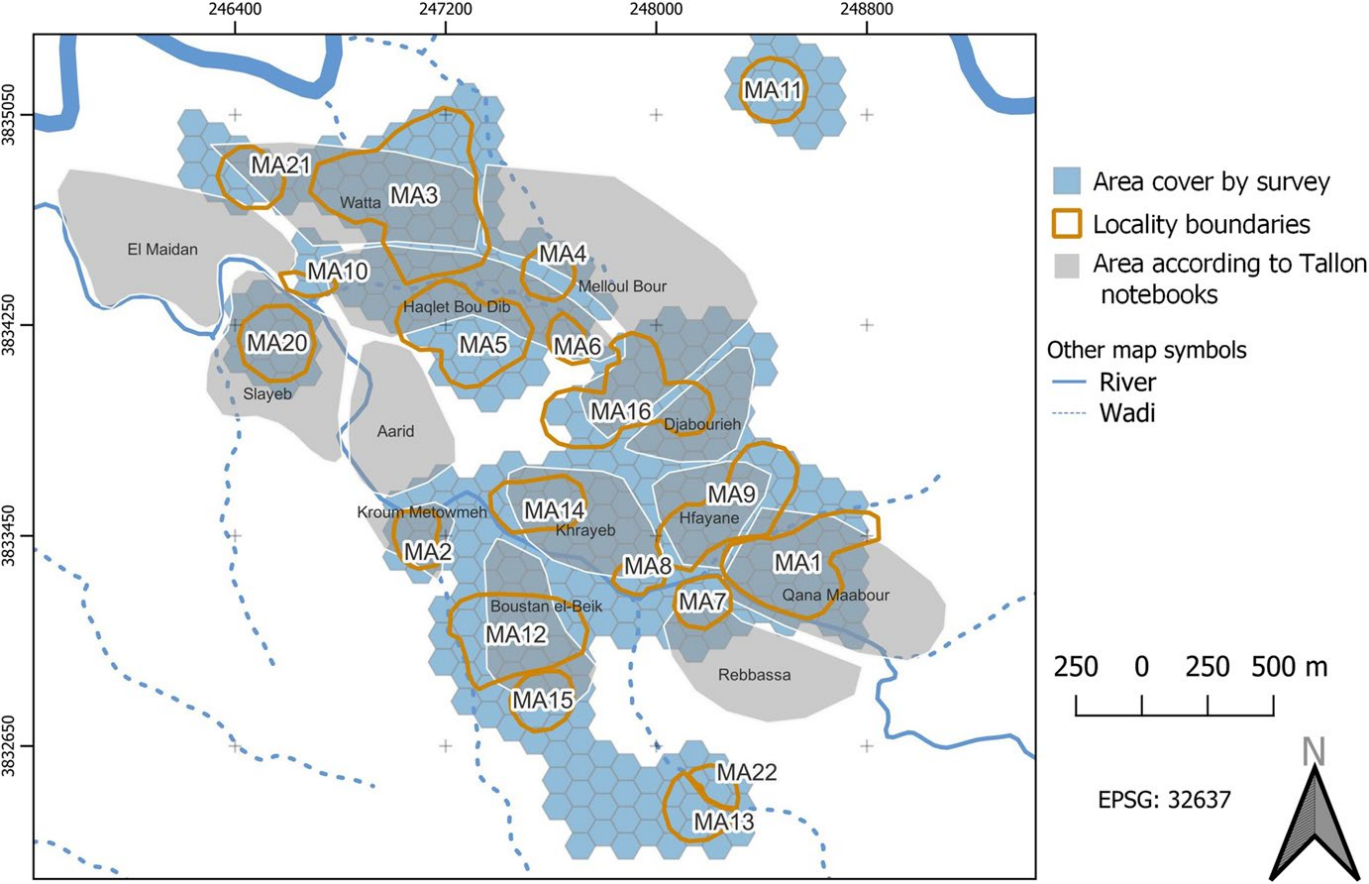
Distribution map of the surveyed areas (in blue), with the numbers of identified sectors (MA) and places defined by M. Tallon between 1959 and 1969 (in grey) (© F. Waldoch)

The megalithic monuments identified in Menjez, within a radius of 500 m around the village, are distributed among several groups, each consisting of 1 to 12 monuments, and occupy sectors (MA1 – MA21) named in reference to associated locations mentioned in M. Tallon’s notebooks between 1958 and 1969 (Fig. 2). In this part of the ‘Akkar, the presence of megalithic monuments is not limited to the territory of Menjez. Several other monuments, the exact number of which is yet to be determined through surveys conducted as part of the MEG-A project, have been observed in surrounding municipalities such as Tlayleh, Kouachra, Fraidis, and Debebbiyeh (Tallon, 1958, 1964; Wygnańska et al., 2022).

The megalithic monuments of Menjez were first subjected to archaeological excavations from the summer of 1961 until 1969, under the supervision of M. Tallon from the Université Saint-Joseph of Beirut. His investigations are documented in five notebooks, preserved at the Université Saint-Joseph and the Museum of Lebanese Prehistory. M. Tallon published a review of his work in Menjez, presenting the results of his excavations between 1961 and 1964 (Tallon, 1964). Following this, for nearly 30 years, no studies were conducted on these monuments. In 1995, T. Steimer took an interest in the ceramic material extracted from M. Tallon’s numerous excavation campaigns. It was also an opportunity to transcribe his notebooks and propose a typology of the megalithic monuments of Menjez (Steimer, 2000).

More recently, these structures have been the focus of several research campaigns. In 2017, a 1-week expertise was initiated by T. Steimer-Herbet, V. Porra-Kuteni, and M. Haïdar-Boustani as part of the project “Valorisation et Protection des dolmens de Menjez,” funded by the British Council – Cultural Protection Fund^1^. The project was an international partnership with the University of Geneva, the Château-Musée of Bélesta, the Museum of Lebanese Prehistory, and the municipality of Menjez. This initiative aligns with a dynamic of enhancing and protecting the megalithic heritage of Menjez, which, like many examples in the Near East, appears to be threatened with disappearance. Indeed, since the 1960s, half of the known monuments in Menjez have vanished. These destructions occurred due to the expansion of the modern village, agricultural activities, and some dolmens were likely used as stone quarries for the construction of village houses.

In 2018, new investigations were launched on these monuments. Twelve of them were cleaned, studied, and 3D-modeled (Steimer-Herbet *et al*., 2018, 2019a, b, 2020). This campaign also provided an opportunity to re-examine the archaeological finds extracted from M. Tallon’s excavations. Subsequently, these 12 monuments were made accessible to the public, and a “Heritage House” was inaugurated in 2019. In parallel, a survey campaign in this part of the ‘Akkar was also conducted in 2018 by Z. Wygnańska and her team^2^.

In addition to the concern regarding the preservation of the megalithic heritage of Menjez, there is a need to better understand these monuments. The pooling of data acquired by two Swiss and Polish archaeological teams gave rise to the MEG-A project – “First megalith builders in the northern Levant” (2022–2025), funded by the Swiss National Science Foundation (FNS)^3^ and the National Science Centre (NCN)^4^, and led respectively by T. Steimer-Herbet and Z. Wygnańska. This collaborative project has two objectives. The first is to define the identity and practices of the megalith builders’ communities in the ‘Akkar. To achieve this, a more precise chronological framework must be established. Considering that Levantine megalithic monuments have been attributed to the 4th and 3rd millennia BCE, thanks in part to excavations over the last 30 years (Braemer *et al*., 2004, 2011; Fernandez-Tresguerres Velasco et Junceda Quintana, 1991; Kerner, 2019; Polcaro et Muniz, 2018; Steimer-Herbet, 2006), the MEG-A project aims to increase the dating of these monuments through the application of various methods. Luminescence dating^5^ is thus being employed within this ongoing research in Menjez, as it has already demonstrated its potential for dating megaliths, notably at Rujm el-Hiri (Freikman et Porat, 2017). It also seeks to establish a cultural framework, determine the ritual identity of these societies, and identify the regional significance of these megalithic ensembles. The second objective is to reconstruct the paleoenvironment of the ‘Akkar and the subsistence strategies of megalithic communities, by determining the natural conditions of the ‘Akkar during the Late Chalcolithic and Early Bronze Age and studying the ways in which these societies adapted.

Architectural study is a major focus of the work carried out by the MEG-A project, as it allows us to grasp the technical and cultural dynamics that characterize these megalithic societies of the ‘Akkar.

## Methods

### Previous Methods Used in Levantine Context

Research into megalithic architecture in the Levant has so far been conducted using standard methods, giving rise to several classifications and typologies of these megalithic monuments (Epstein, 1985; Fraser, 2018, 2022; Joussaume, 1985; Steimer-Herbet, 2004, 2022; Stekelis, 1961; Zohar, 1992). Nevertheless, it is worth noting that some researchers have already proposed an in-depth study of megalithic buildings in the Levant. At Khirbet al-Umbashi, several issues were addressed, including the supply of raw materials, in this case basalt, with a focus on quarrying, transport, and storage of the blocks and their layout within the architecture (Braemer *et al*., 2004). Building methods were also studied, with a particular focus on corbelled roofs covering the funerary and domestic structures identified at Khirbet al-Umbashi (Braemer et Sorin, 2010).

Usual methods are based on a study of the plans of these monuments, which implies to considering them in 2D only. In contrast, the methodology we are applying to Menjez, based on buildings archaeology, enables us to consider the monument in its entirety. We thus move from a 2D plan view to a 3D perception. The difference lies in the fact that the monument’s elevations are considered in our archeological reading (Cousseau, 2020). Like subsoil archaeology, the constructed units of elevated buildings are conceived as stratigraphic units. In this way, buildings archaeology and subsoil archaeology are based on the same principles and employ the same methods (Reveyron, 2002, p.6).

### Western European Methods Background

Buildings archaeology is a discipline that emerged in the 1980s (Prigent, 1989) and developed from the restoration of monuments located in historic city centers of major metropolises (Reveyron, 2002, p.5). Traditionally used to study medieval and modern buildings, it has since evolved to include the study of built monuments of all periods. Buildings archaeology seeks to break down the architectural phasing of a building and reconstruct the various architectural projects that shaped it (Laporte, 2016, 2022). In this way, the application of this method of archaeological investigation reveals the history of a monument (Boto-Varela *et al*., 2012, p.330). The application of buildings archaeology tools to Neolithic monuments described as megalithic was first undertaken on almost entirely dry-stone built tumulus C of Péré at Prissé-la-Charrière (Deux-Sèvres, France) (Cousseau, 2015; Laporte *et al*., 2014, 2021), based on the pooled skills of two researchers, a medieval building archaeologist (I. Parron) and a specialist of megalithic architectures (L. Laporte) (Laporte & Parron-Kontis, 2010). This first experiment proved conclusive, with significant results on the application of stratigraphical reading on dry-stone masonry. “The methods of buildings archaeology applied to the megalithic constructions of Western France (Cousseau, 2020, 2023) have (…) allowed to highlight all the technicality implemented…” (Laporte *et al*., 2020, p.13), starting by enabling researchers to restitute the various actions of the builders, identifying the different skills needed, shedding light on the successive architectural projects, and the organization of work on such sites. The contribution of such a methodology to the advancement of research on monumentality is therefore considerable for our understanding of megalithic societies, whose technical and cognitive expertise is anything but rudimentary and appears more complex than previously imagined (Laporte *et al*., 2020).

The challenge was then to multiply the applications of this new methodological approach to other megalithic monuments with the same characteristics and thus determine the validity of this method (Laporte *et al*., 2014, p.176–177). One of the co-authors (F. Cousseau) was the first to multiply the application of these new tools to the study of Western European megalithic buildings, by combining them with a geoarchaeological approach (Cousseau, 2023)^6^. Thus, he was also the first to propose a reading of the geological and geomorphological characteristics of the monoliths that make up the internal space of Western European megalithic monuments, based on work conducted, among others, on the alignments of Carnac (Brittany, France) (Mens, 2008; Mens et Large, 2009; Mens *et al*., 2022; Sellier, 1991, 2013, 2018). This practice has since been democratized in Western Europe and has been applied to Neolithic megalithic monuments which take the form of large burial mounds made largely of dry-stone walls, formed from blocks of small module (Cousseau, 2023; Gouézin, 2022; Gouézin *et al.*, 2019; Linares-Catela, 2017; van der Reijden, 2020). The architectural features of these monuments make this method easy to adapt and are now well-established within European Neolithic specialists (Laporte *et al*., 2014, p.188).

As I. Parron-Kontis and N. Reveyron noted, buildings archaeology has made it possible to deliver “a large body of documentation that reveals a great diversity of methodological approaches: archaeologists must constantly adapt to the objects being studied” (Parron-Kontis et Reveyron, 2005, p.7). The method proposed by buildings archaeology can therefore be modeled according to the contexts and buildings studied. The challenge for us is to adapt this new methodological approach to the Levantine megalithic context and its specificities and thus multiply its application beyond the Western European context, starting with the megaliths of ‘Akkar and in particular those of Menjez. This new way of looking at megalithic monuments in the Levant will enable us to offer new keys to reading and understanding the megalithic phenomenon in this part of the world, following the example of what has been undertaken in Western Europe and the encouraging results obtained.

### Adaptation to Menjez’s Megaliths: The Levantine Megalithic Building Techniques

Proposing this new methodological approach to the study of Levantine megalithic monuments was motivated by the need to increase our knowledge of the societies associated with them. Megalithic phenomenon is by no means uniform and homogeneous; it takes many diverse forms. There are many differences between Western European and Levantine megalithic architecture, starting with building techniques. In the Levant, and particularly in Menjez, we are dealing with monuments built mainly from large-module monoliths and standing stones. We also come across dry-stone walls, but once again, the stone module used is not the same, and the blocks are generally larger than those used to build the monuments studied in Western Europe using this method. We must deal with blocks of very different sizes and patterns and therefore propose an innovative and a specific adaptation of the method to Menjez monuments. The geological context and its geomorphological development are also original, and this plays a major role in megalithic construction. Indeed, the lithological characteristics of the local rock will condition the way in which monuments are built. All this means that we need to propose an adapted version of the method used in Western Europe and develop new methodological tools and study criteria.

Consequently, for the first time in the Levant, at Menjez, we have adapted a method combining buildings archaeology and geoarchaeology to the specific architectural features of the megalithic monuments studied and to the particular geological context of this part of the Lebanese ‘Akkar. We then developed our method according to three levels of reading grids. The first grid consists in highlighting the intrinsic characteristics of the studied monument. It is essential to put into perspective the image we have of the megalithic tomb in the Levant. Known as a dolmen, this funerary monument generally takes the form of a trilithon and consists of the assembly of large blocks. Nevertheless, according to the terminology used in Western Europe (Cousseau, 2023; Gouézin, 2022; Laporte *et al*., 2014), the term “dolmen” simply refers to the internal space of the original monument, mentioned as the sepulchral space in some publications, which in many cases corresponds to the only element surviving over time (Cousseau, 2023, pp.xiii-xiv). It is therefore likely that these monuments were originally larger and more complex constructions, with different spaces interconnecting, each having its own architectural identity. This leads to our second reading grid, which is based on principles developed in the field of buildings archaeology. The aim is to break down the monument into several building units and establish the architectural phasing that defines the structures under study, by restoring the operating chains that condition their construction. The third grid, finally, consists of proposing a geo-archaeological reading of the material used for the construction of the monuments, namely the monoliths formed from the surrounding basalt. Taken together, these different approaches inspired by Western European megalithic monuments offer an in-depth interpretation of the megalithic architecture of Menjez. Thus, we aim to multiply methodological tools to provide new insights into Levantine megalithic monuments.

### First Reading Level: Plan and Volume

The first level consists of identifying the volumes of the structure, describing the different spaces it comprises, and defining their functions. To do this, we had to establish a terminology adapted to the specific architectural features of the Menjez megalithic monuments. We distinguish between the chamber, which corresponds to the monument’s internal sepulchral space and which can be associated with a corridor; the crown, which takes the form of a dry-stone wall serving as a rear support for the chamber walls; the platform, also made up of an external dry-stone wall combined with a filling of small blocks, which can replace the crown; the enclosure, which forms the limits of the monument’s space, with dry-stone walls without associated filling; and the ring, which consists of a pile of stones of any module arranged in a circle around a monument.

**Fig. 3:**
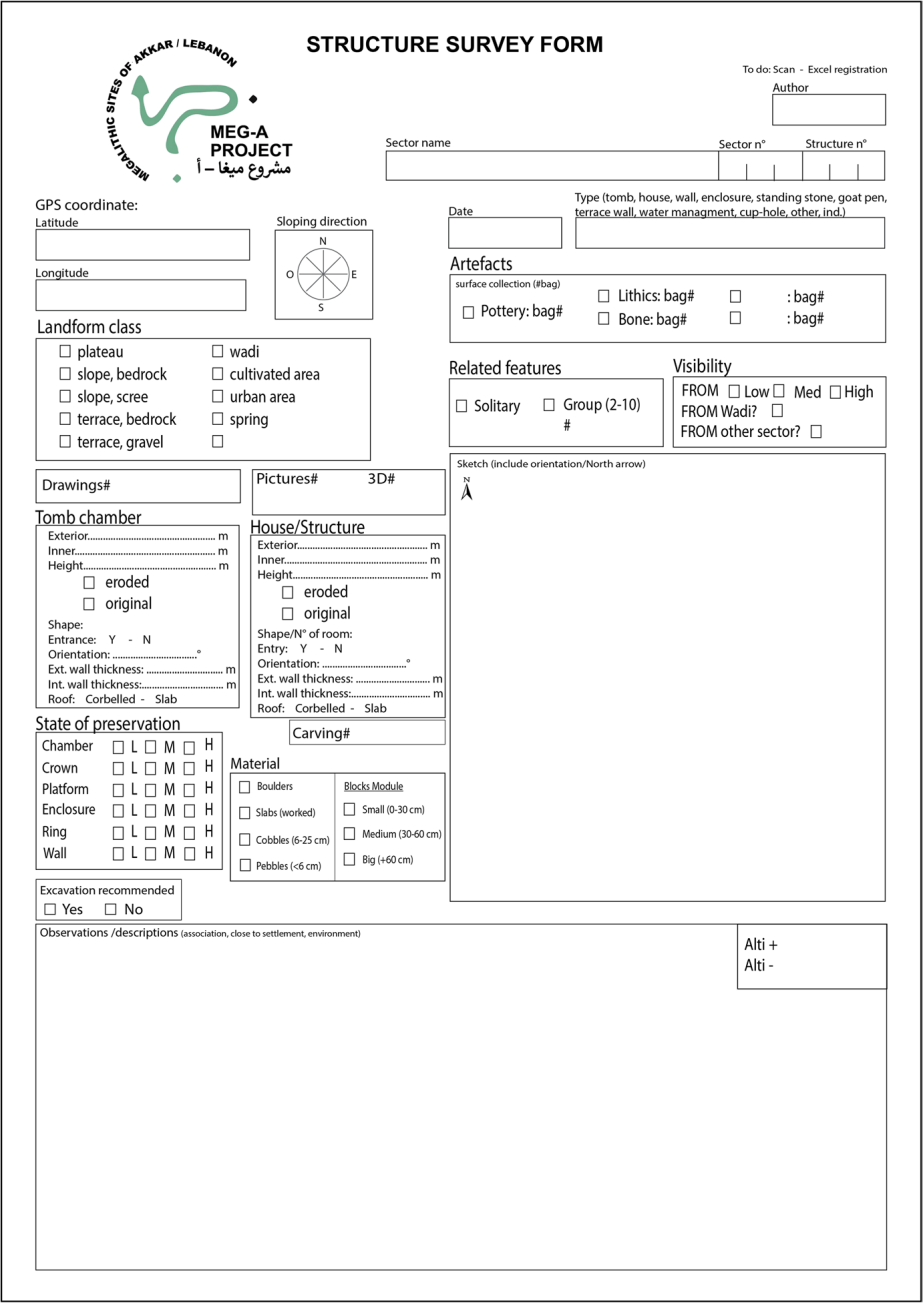
Structure survey form (© MEG-A)

This initial step allows us to propose a classification of the megalithic monuments of Menjez, by determining whether recurring forms can be observed and, consequently, whether common practices are attested. To achieve this, we examine the general shape of these monuments, their dimensions, orientation, and their state of preservation, which conditions our reading. All these elements are then observed and recorded in a database (Fig. 3). We also take into consideration the environment and topographical location of the monument. The latter plays a crucial role in the design of the megalithic monument, as architecture is influenced by its immediate environment, leading to the development of technical and innovative means obliging builders to implement specific adjustments.

### Second Reading Level: Operating Chains and Architectural Phasing

A three-dimensional photogrammetric survey was conducted on all megalithic monuments. This 3D model serves as a complementary tool, enabling us to obtain a digital copy of the studied structures and produce the necessary documentation. Thus, ground plans, profiles, and sections of these monuments were generated to facilitate the reading of the various architectural components. This allows for the dissection and decomposition of the monument, which is no longer considered as a monolithic entity but as an assembly of several constructed units interconnecting with each other (Mens & Large, 2009, p.43).

**Fig. 4:**
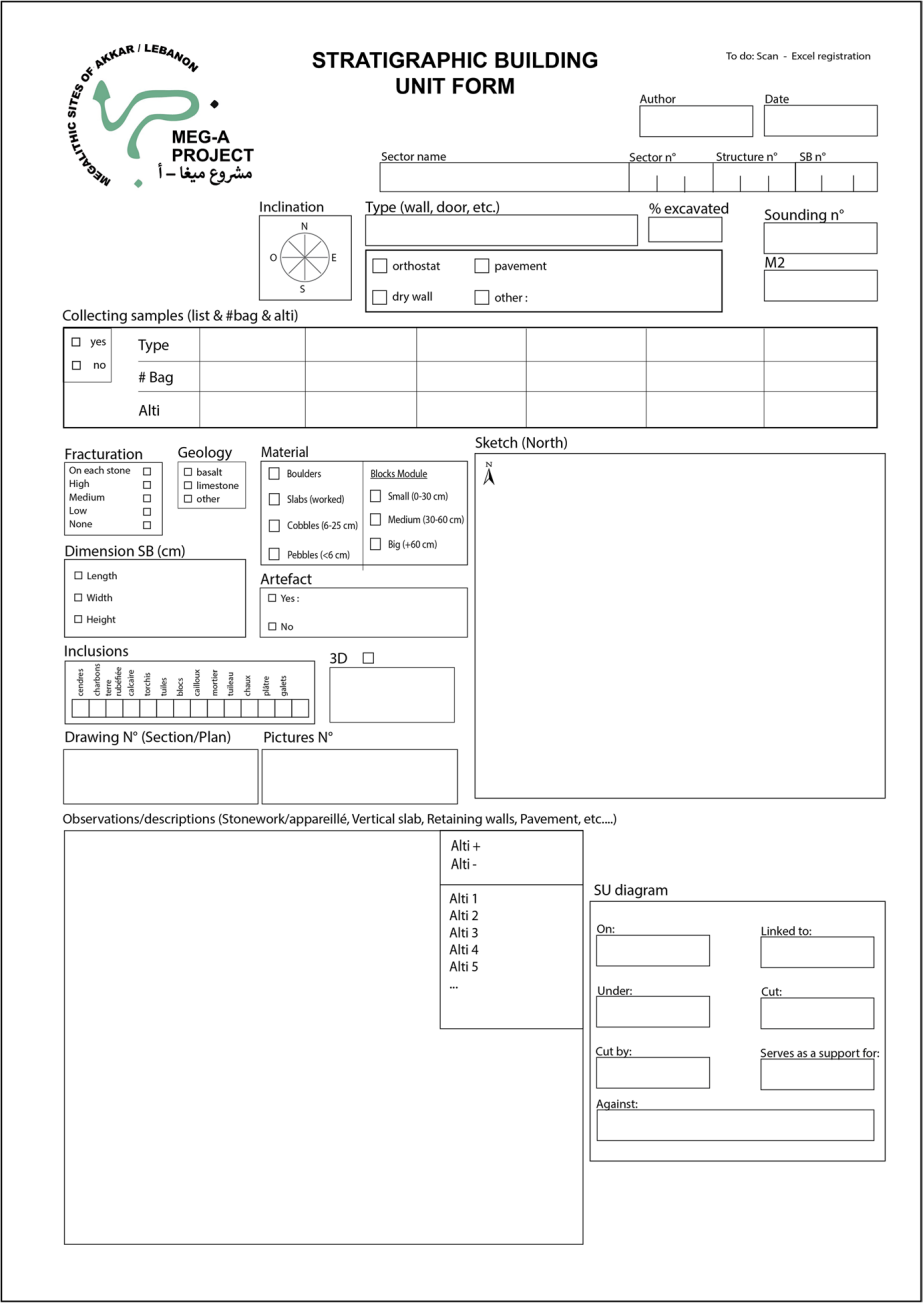
Stratigraphic building unit form (© MEG-A)

The second level of reading involves defining *stratigraphic building units* (Fig. 4). The constructed units of elevated buildings are conceived as stratigraphic units (Reveyron, 2002, p.6). The goal is to identify discontinuities and breaks in construction and divide the structure into several building units. The megalithic monument is no longer considered as an indivisible whole but as an assembly of several building units, leaning against or superimposed on one another. As part of Menjez, we have established various criteria to analyze these different building units: their masonry, the building material used, defining three block modules^7^ and their geological sourcing, as well as their functions.

Through the deconstructing of these monuments into different units, we are able to propose a relative chronology of the construction of these structures, in the manner of a stratigraphic diagram, in which the various units succeed one another (Cousseau, 2023, p.99). This stratigraphic sequencing of the building allows us to reconstruct the architectural phasing of these structures (Laporte *et al*., 2020, p.16 & Figs. 10, 11, 12, and 13). The goal is to understand how the megalithic monument evolved over time. To achieve this, we distinguish building stages and building phases. These terms refer to two distinct time scales. Building stages imply a short time period and are applied to a single architectural project. Building phases, meanwhile, imply one or more significant breaks and therefore a longer period of time and distinct architectural projects (Cousseau, 2023, pp.55–56). This includes various modifications, additions, and destructions visible in the stratigraphic sequence of the structure and occurring once the initial architectural project is completed (Laporte, 2010). As pointed out by Gouézin (2022, p.10), in many cases, megalithic monuments are the result of a complexity of architectural projects, reflecting distinct building techniques and motivations (Laporte *et al*., 2021; Linares-Catela, 2021, 2022; Porqueddu *et al*., 2023).

By applying this methodological approach, we can address several questions. First, we can restore the various gestures used by the builders in the construction of these megalithic tombs. We can therefore determine whether these actions are repeated from one monument to the next and establish whether there is a certain consistency in the construction process of these monuments or whether there are different ways of conceiving them. Second, we can identify whether changes are noticeable in the architecture and highlight various technical innovations observable within a monument.

### Third Reading Level: The Study of Monoliths

**Fig. 5:**
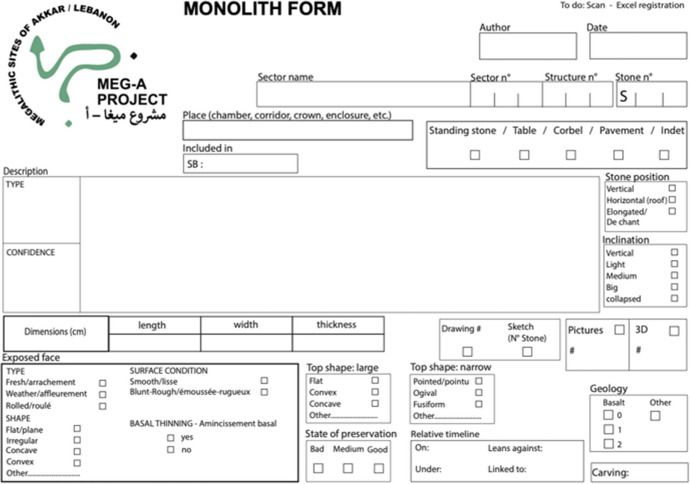
Monolith form (© MEG-A)

The third level focuses on the monoliths that form the main material of these structures (Fig. 5). Detailed observation of these blocks provides us with numerous insights into the technological and symbolic context, through a geo-archaeological approach, and represents a significant part of the work carried out in Menjez.

We focused our attention on the orthostats, the paving blocks, the covering blocks (tables and corbels), and the entrance slabs forming the walls of the corridor. Some of the blocks lying outside the structure’s space, when the monument collapsed or was disturbed, were also integrated into the analysis.

Observing the monoliths informs us about the patterns and rhythms sought during the placement of blocks within the structure. This enhances our understanding of the symbolic dimension expressed in the architecture. In general, the chamber contains the most elements in this regard. These observations have led to the affirmation that there is no randomness in the arrangement of orthostats. To establish this conclusion, our study of the monoliths relies on the observation of their architectural, geomorphological, and geological characteristics, which seem to condition their placement within the monument.

#### Architectural Features of the Monolith

To begin our study of monoliths, we defined four criteria of analysis relating to the form and function of the block under study. The first criterion concerns the placement of the monolith within the monument (chamber, corridor, cover, crown, platform, enclosure, ring), as well as the category to which it belongs (orthostat/standing stone, paving block, slab, cover table, corbel, foundation block, *etc*.). This first criterion allows us to assign a function to the monolith. In the same time, we also examine the general shape of the monolith, which determines the block’s location within the monument and thus defines its function. The second criterion is based on the dimensions of the block, including its length, width, and thickness, aiming to establish if a connection can be made with the block’s function. The third criterion is to determine the position of the block (vertical, on edge, or horizontal) and its inclination. The last criterion concerns the shape of the top of the monolith. We distinguish between two groups: wide tops and narrow tops. Within these groups, we define various shapes: flat, concave, or convex for the first, and pointed, ogival, or fusiform for the second. These last two criteria are almost exclusively related to the orthostats that make up the walls of the chamber. They enable us to determine whether patterns and rhythms are perceptible in megalithic architecture.

##### Geomorphology and *Mental Refitting*

The observation of the geomorphological characteristics of the monoliths relies on a method developed by Sellier (1991, 2013, 2018, 2023). His method “comprises four levels of analysis related to the general shape of the blocks used as menhirs, the morphology of the sides (including weathered faces and fresh faces), the characterization of pre-megalithic or post-megalithic forms, and the surface states of the menhirs” (2018, p.289). In the context of Menjez, we have adopted three of these analysis criteria. The first involves examining the morphology of the monolith’s main faces, distinguishing between flat, concave, convex, or irregular shapes. The second criterion is based on identifying observable forms of erosion on the so-called exposed face of the monoliths, which corresponds to the face that was visible during the use of the tomb. There are two types of erosion forms: pre-megalithic and post-megalithic. Pre-megalithic erosion forms provide information about the origin of the blocks used as monoliths in the monuments. Post-megalithic erosion forms inform us about erosion dynamics, with the construction of the monument or the collapse of the monument, exposing certain blocks initially protected from erosion, serving as temporal markers (Sellier, 2018, p.289). The third criterion involves defining the exposed face and its type, *i.e*., whether it is a fresh face or a weathered face. A fresh face corresponds to the surface of the block that was attached to the bedrock substrate before its extraction, while a weathered face refers to the surface of the block that was exposed and outcropped or which emerged from the ground. Fresh faces generally have angular edges and rather regular surfaces, unlike weathered faces that appear as irregular surfaces with smoothed angles, having undergone erosion (Sellier, 2018, p.295). The identification of erosion forms also serves as a good indicator to recognize the type of face under consideration.

We paid particular attention to face identification, especially for the exposed face of the monolith. More broadly, all faces of a monolith were observed whenever possible in the field. This allowed us to determine whether the monolith was exclusively made up of weathered faces, fresh faces, or a combination of both.

**Fig. 6:**
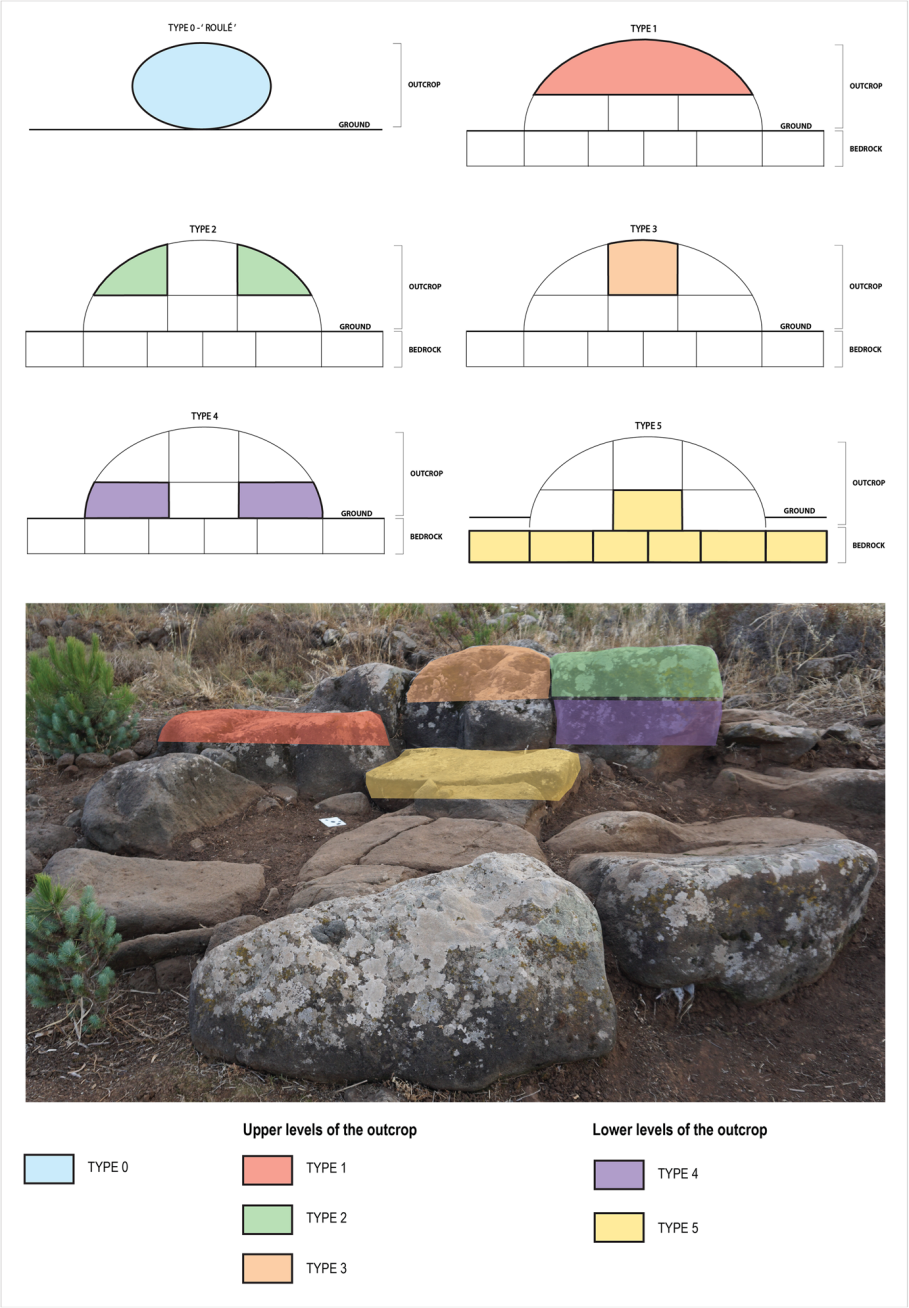
Types of monoliths according to the mental refitting method, defined by Mens (2008), illustrated on a basalt outcrop used as a quarry - Kroum Metowmeh - MA2

Based on this geomorphological approach, Mens (2008) has established a typology of the different monoliths that make up a megalithic monument or complex. This method, which E. Mens calls *mental refitting*, consists in mentally resituating the block within the rock outcrop and thus knowing its original position. This allows for establishing a relative chronology of the extraction work conducted and therefore determining the operating chain of the monolith, from its supply in raw material form to its installation within the structure. This method is based on a principle developed by specialists in flint knapping, who in attempting to understand how stone tools were made, seek to reposition flakes, and thus apprehend the executed gestures (Mens *et al*., 2022, p.5). The same process can be applied to rocky outcrops that provided the raw materials for the construction of megalithic monuments. Based on this observation, E. Mens defined 5 types of monoliths. Types 1, 2, and 3 correspond to blocks that come from the surface levels of the original outcrop, while types 4 and 5 correspond to blocks from the deepest levels of the outcrop and are largely made up of so-called fresh faces. This typology has been developed notably from the granite which constitutes the raw material of the menhirs’ alignments in Carnac. In Menjez, we deal with basaltic outcrops, material for which *mental refitting* method is adaptable and transferable. To do this, we added type T0, which corresponds to an outcrop block entirely detached from the substrate. The application of this method is an essential part of our study as it allows us to reconstruct the supply of raw materials and to understand the mechanisms of block management (Fig. 6).

##### Geological Feature: Menjez Basalts

Special attention has been given to the geological criterion. The local geology is predominantly composed of basaltic substrate (Abdel-Rahman & Nassar, 2004; Shaban, 2010), resulting from volcanic activity during the Pliocene period (5.332 million–2.588 million years ago), during an orogenic period corresponding to a folding phase. It belongs to what is known as the Homs basaltic nappe, which extends across this part of the Syrian-Lebanese ‘Akkar. This basaltic field takes the form of several layers of basalt, some 200 m thick, corresponding to a succession of basaltic lava flows. The Menjez region lies at the junction of the Yammouneh transform fault and the Ghab transform fault, which are part of the Dead Sea fault system, forming the boundary between the Arabian and African plates, and the Levantine sub-plate. This situation makes the ‘Akkar region highly vulnerable to seismic and tectonic movements, with overlaps being frequent. This plate tectonics has fractured the thick basaltic layer, creating rectilinear joints visible in outcrops. Curved joints formed from lava flows are also visible in the outcrops. These different fractures facilitate extraction and condition the shape of the blocks used as building material for the megalithic monuments in Menjez and the surrounding area (Steimer-Herbet *et al*., 2022; Wygnańska *et al*., 2023). Indeed, many monoliths used in the construction of Menjez monuments show no signs of cutting or shaping.

Geological observations played a crucial role in the study of European Neolithic megalith sites, particularly to address the question of raw material sourcing, by identifying the supply chain and determining the distance between the place of acquisition of the blocks and the megalithic construction site (Mens *et al*., 2022, p.3). The researchers who used this criterion to study monoliths were then interested in the petrography and natural color of the megaliths, in order to highlight certain chromatic effects. In examples from the Atlantic coast, there is a geological variation in the internal space of megalithic monuments, which creates rhythms in the choice of orthostats, through plays of color and texture (Cousseau, 2023, p.37; Cummings, 2002; L’Helgouach, 1995; Linares-Catela *et al*., 2023; Scarre, 2004). “The interplay of natural colors and petrography are two elements that constitute a major part of the architectural discourse in a number of burial chambers on the Atlantic coast.” (Mens *et al*., 2022, p.7).

**Fig. 7:**
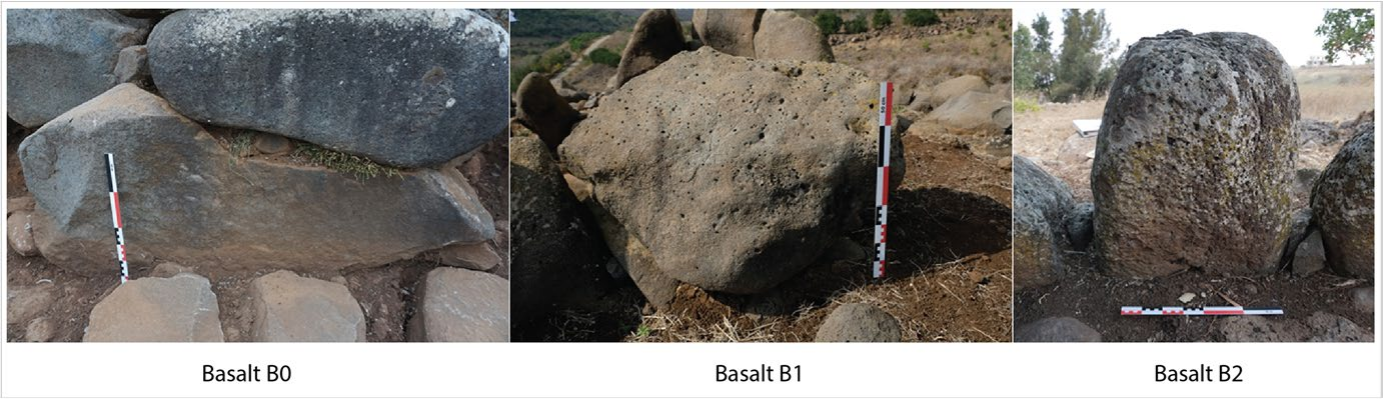
Basalt classification according to vesicularity

We had to adapt this method to the particular geological conditions in Menjez. The first step was to carry out observations on the lithological characteristics of the blocks and outcrops. Contrary to European examples, no geological or color variations were observed among the monoliths of the Menjez monuments. Only basalt is used, due to the particular geology of the region. The geological criterion in our study is therefore based on textural variation. We identified and characterized different basalts, based on the degree of “vesicularity” that characterizes Menjez basalt. This “vesicularity” was defined according to the volume proportion of vesicles observed on the monoliths. These vesicles are the result of oxygen bubbles which, during the solidification of the basalt, rose to the surface and gave way to characteristic small perforations. Three types of basalt have thus been defined: B0, B1, and B2 (Fig. 7). B0 basalt is a “non-vesicular” basalt with a smooth surface. It is the densest basalt and the least exposed to erosion. Basalt B1 is moderately vesicular, with vesicles measuring less than 1 cm. Basalt B2, on the other hand, is characterized by numerous vesicles on the surface, generally measuring over 1 cm. The surface of the basalt corresponding to this latter type is generally quite rough. Identifying and defining the type of basalt enables us not only to discuss the supply of blocks, but also to shed light on the play of textures sought during the construction of monuments.

The application of the geological criterion is essential, since the lithological characteristics of a rock will determine the shape of the future monoliths (Sellier, 2018, p.294). This enables us to identify where the blocks were acquired and to determine the basalt flow from which they were extracted.

Considering geology and geomorphology allows us to contemplate and study the funerary and sepulchral space in its entirety, from the natural environment in which it is located to the interior of the tomb.

The combination of methods developed by D. Sellier and E. Mens enables us to apprehend two factors. First, we can reconstruct an overview of what the pre-megalithic landscape might have looked like—before the extraction and acquisition of basalt blocks used in the construction of megalithic tombs—and therefore account for this pre-megalithic landscape. Second, it provides insights into the erosion dynamics that have acted on the basalt blocks over time. Finally, when combined with the study of the construction, these observations highlight the various patterns and rhythms sought by the builders of megalithic monuments.

##### Case Studies

**Fig. 8:**
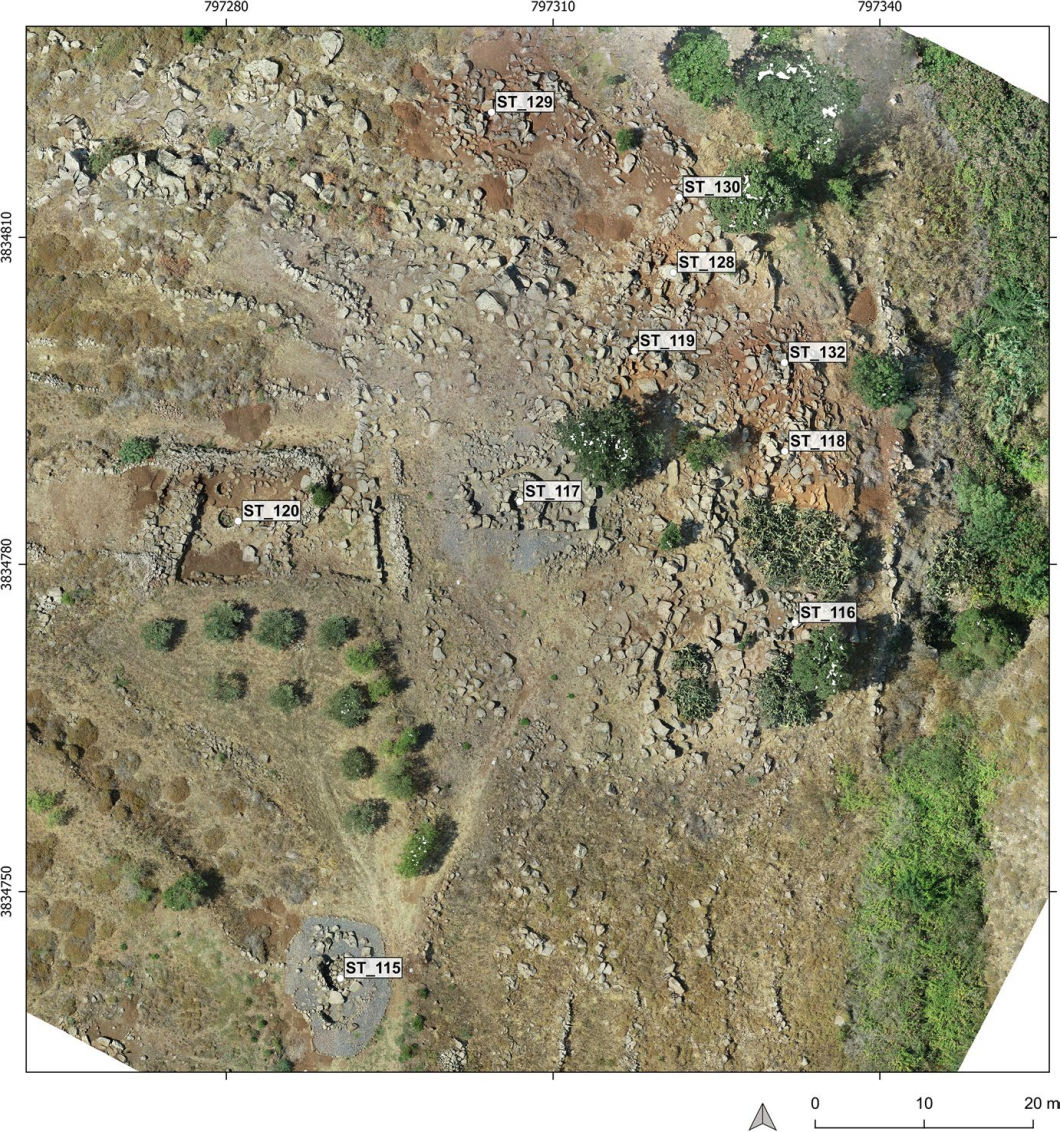
Map of Kroum Metowmeh area - MA2

We applied our methodology to 24 monuments in Menjez. Here, we present two tombs, ST_118 and ST_129. These monuments are located in the MA2 sector at a place called Kroum Metowmeh, situated southwest of the village of Menjez (Fig. 2). They are part of a cemetery consisting of eight megalithic tombs (ST_115 to ST_119 and ST_128, ST_129, ST_132), associated with a structure interpreted as a double-apse house (ST_120), cyclopean walls cutting through the house, and several terrace walls (Fig. 8). The contemporaneity between the dwelling, walls, and funerary monuments however remains to be demonstrated in future campaigns.

The topography of the Kroum Metowmeh sector (MA2) comprises a plateau, on which we identified two large megalithic tombs and the double-apse house. It also includes a steep slope plunging towards the Wadi Moungez to the north, on which the six other megalithic tombs are located. This slope has been terraced, and it is precisely on these terraces that some of the megalithic monuments have been built.

##### Tomb ST_118

**Fig. 9:**
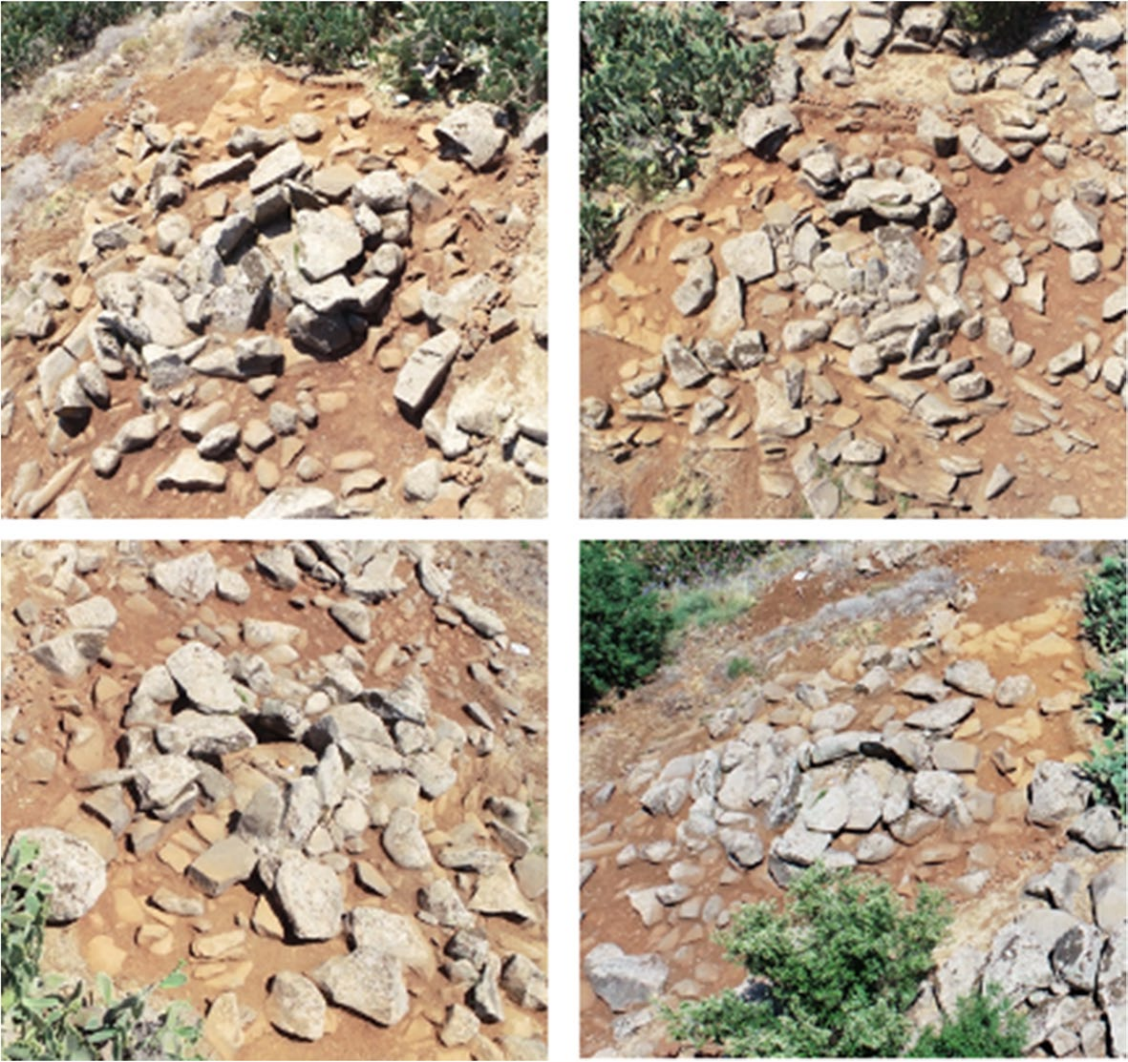
UAV views of megalithic monument ST_118 (top left: from the north; top right: from the east; bottom left: from the south; bottom right: from the west) (© DGA). The internal diameter of the chamber is of 2.30 m from north to south and 1.90 m from east to west, and the external diameter of the crown is of 5.00 m north-south and 4.10 m east-west

Tomb ST_118 is one of the best-preserved tombs in the Kroum Metowmeh sector (MA2) (Fig. 9). It is located on the sloping terraced ground and rests upon one of these terraces. It also leans against one of the terrace walls. However, its eastern part, towards the slope, is less well-preserved, especially regarding the crown space, whose upper courses have collapsed. This megalithic tomb appears to be part of a row of tombs, erected on the same terrace and consisting of ST_116, ST_118, ST_132, and possibly another tomb located under a cactus between ST_116 and ST_118.

#### First Level: Plan and Volume

**Fig. 10:**
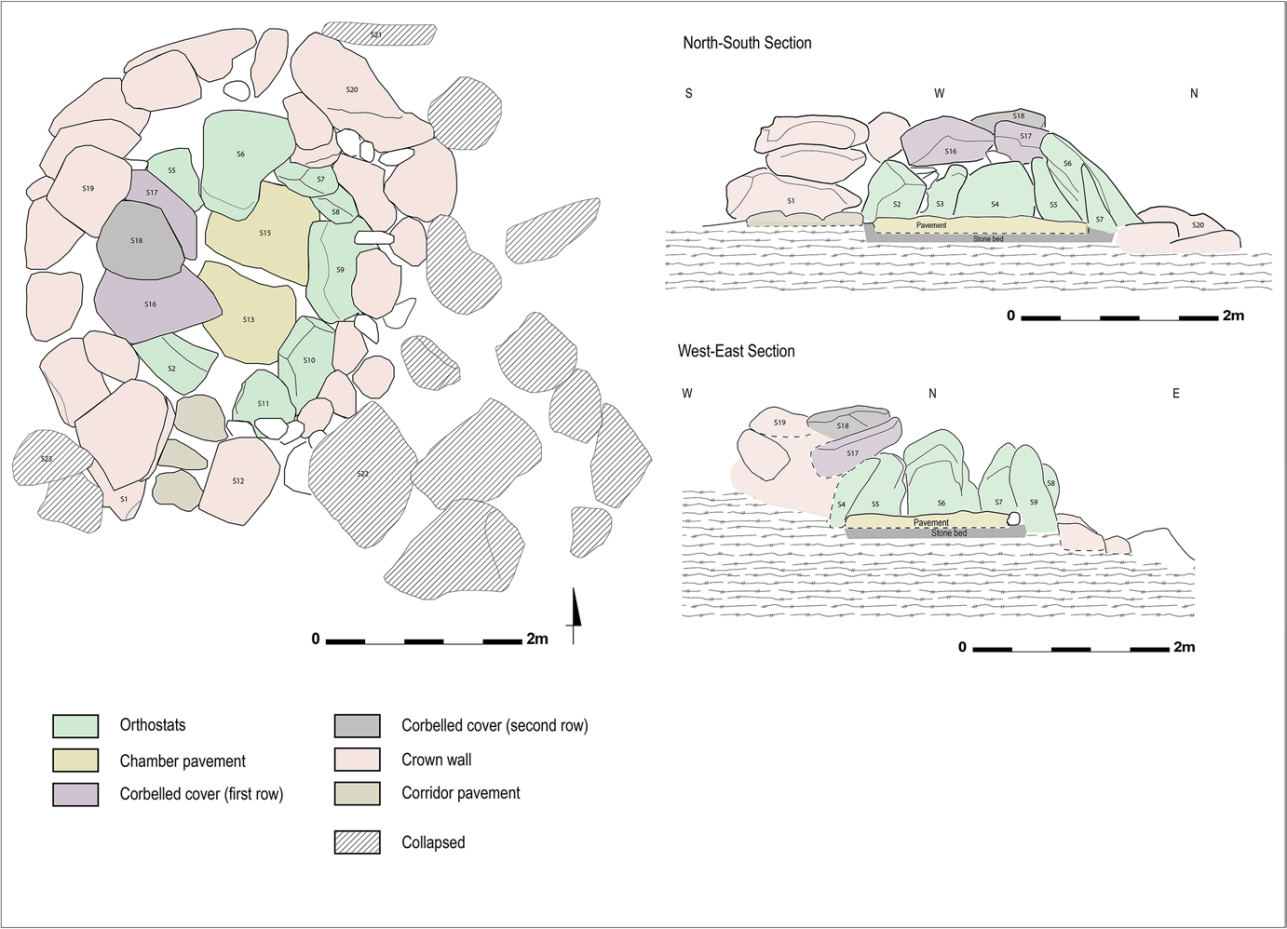
Spatial distribution of ST_118 components (link to 3D model - Sketchfab: https://skfb.ly/oTrBn)

**Fig. 11:**
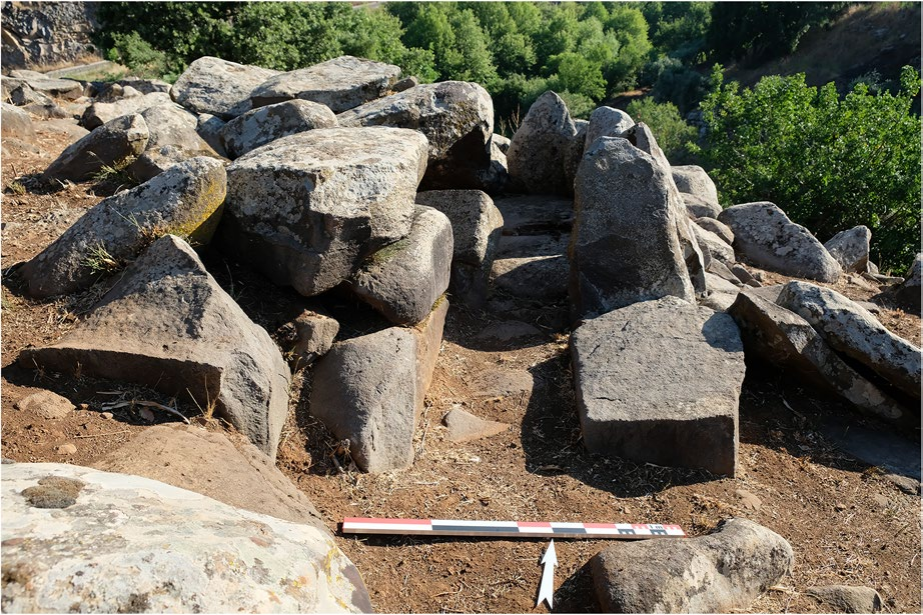
View on ST_118 from the south

This megalithic tomb consists of a circular chamber with an internal diameter of 2.30 m north-south and 1.90 m east-west, delimited by 10 orthostats still in place (Figs. 10 and 11). These orthostats are supported at the front by the chamber’s pavement and at the rear by the crown wall. The chamber’s pavement is made up of three massive blocks resting on a stone bed, a layer of small stones laid as a foundation for the chamber space. However, this foundation has not been identified in the crown space, where the foundation blocks rest directly on the substrate.

Originally, the orthostats supported blocks that formed the chamber’s corbelled roof. Three corbels, the horizontally arranged blocks forming this roof, are still in place. Two of them formed the first row of corbels, while the third, resting above the first two, corresponds to the second row. These corbels are supported on one hand by the chamber orthostats and on the other hand by the crown wall.

**Fig. 12:**
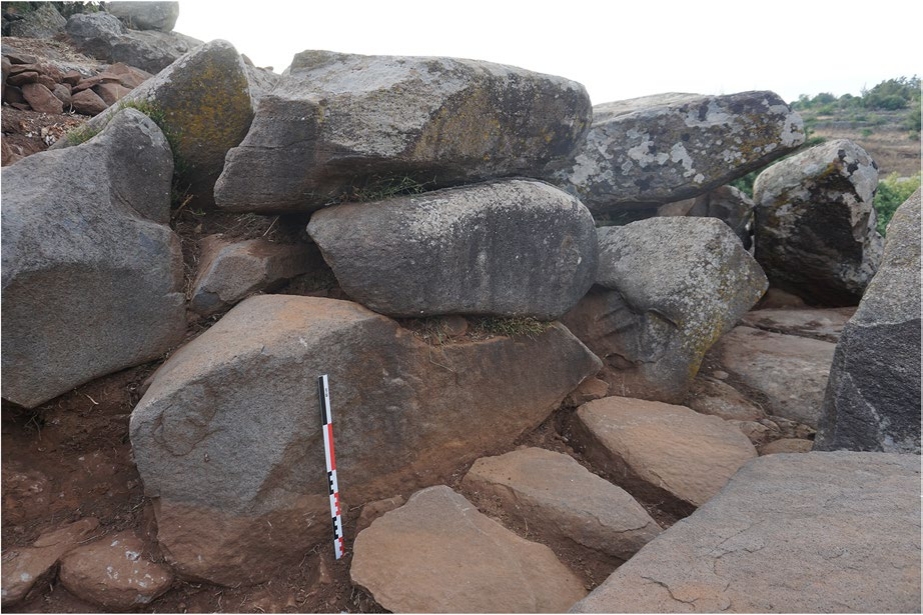
View on the western wall of ST_118’s corridor, from the south-east

The chamber is accessible through a corridor, 1.50 m long, oriented south-southwest. The western wall of the corridor is well-preserved, with three courses formed by large horizontally arranged slabs (Fig. 12). The eastern wall, however, is less well-preserved, as only one large horizontal slab forming the lower course remains in place. On the floor, the corridor’s pavement was uncovered during the tomb’s cleaning. It consists of three small slabs arranged along the corridor walls, integrating into the crown surrounding the chamber. No specific arrangement was discovered at the entrance of the ST_118 chamber. The transitional space between the corridor and the chamber seems to have been filled with small stones, and thus there is no connection between the corridor’s pavement and the chamber’s pavement.

The chamber is surrounded by a circular crown, preserved over a diameter of 5.00 m north-south and 4.10 m east-west. This crown takes the form of a single-face wall made of large module blocks. To the west, two to three courses of blocks have been preserved, while to the north and east, only the foundation course remains. To the north and west, the foundation course consists of massive blocks resting directly on the substrate. These blocks must have required considerable effort to place. As previously mentioned, the crown served as support for the chamber’s orthostats, as well as the rows of corbels forming the chamber’s roof. The orthostats of ST_118 were not installed in a pit, which is a peculiarity of the Menjez megalithic monuments. The orthostats are simply supported to the front by the pavement, either directly or by means of small wedging stones, and at the rear by the crown. In the eastern part of the crown, some of the foundation blocks bear traces of shaping on their upper western edges, *i.e*., against the orthostats of the chamber’s eastern part. This indicates that these blocks have been worked to fit the morphology of the orthostats, to ensure better wedging and stability for the chamber.

#### Second Level: Operating Chains and Architectural Phasing

**Fig. 13:**
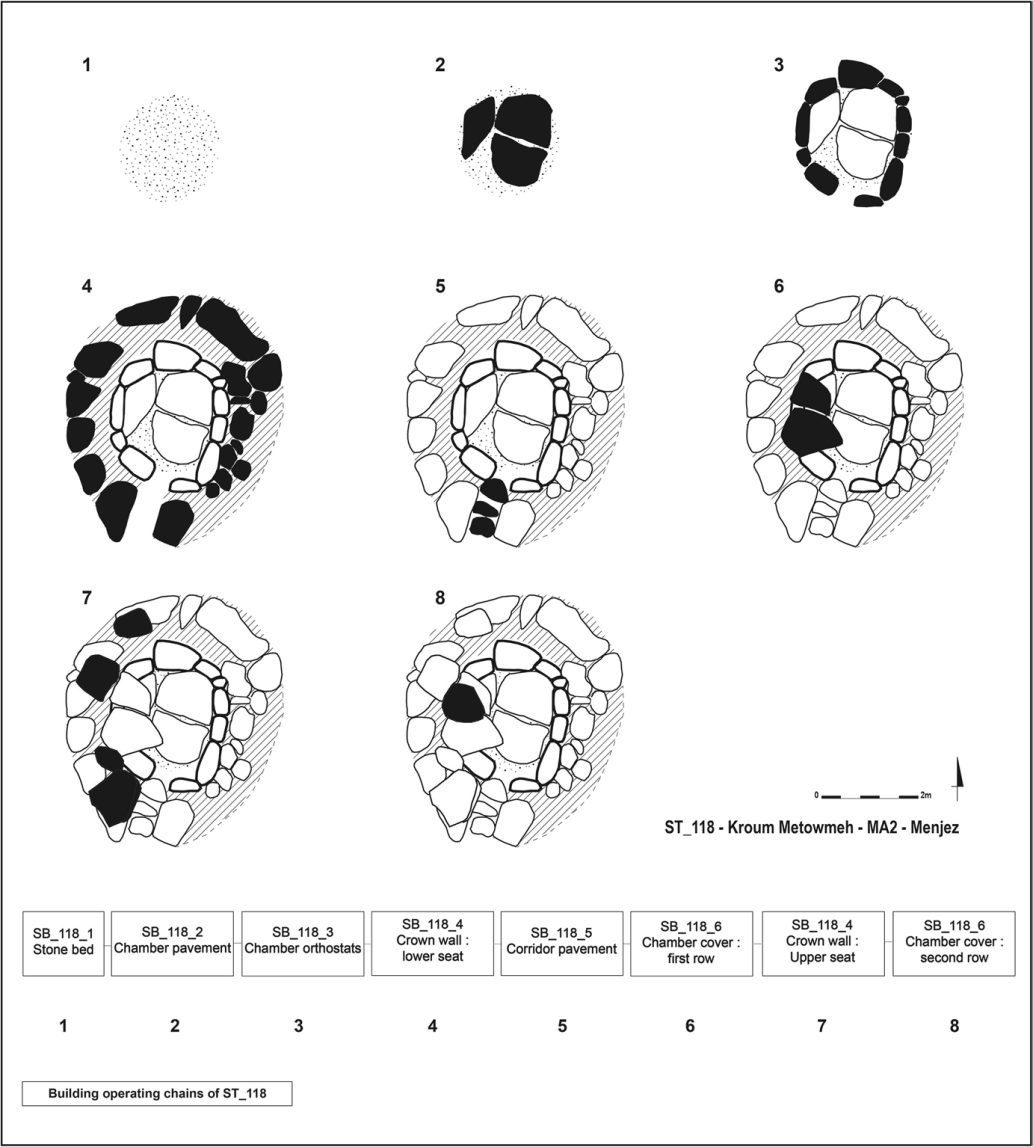
Building sequence with stratigraphic building units of ST_118

Tomb ST_118 appears to have been built in a single phase and must therefore correspond to a single architectural project (Fig. 13). The interpretation of the building sequence of ST_118 is not complete, due to the state of preservation of the monument. Therefore, for what can be seen today, the building sequence can be split into eight building stages, from ground preparation to completion of the architectural project.

Initially, the stone bed was laid on the bedrock surface at the location of the future chamber. This process primarily serves to level the ground before accommodating the rest of the structure. The chamber’s pavement blocks were then arranged above this stone bed. The gaps left between these blocks were then filled with tamping stones.

The orthostats were then placed all around the pavement and the stone bed. Small wedging stones were arranged between the base of the orthostats and the pavement blocks to ensure the stability of the chamber walls. Once the orthostats were positioned and supported at the front, the crown foundation was laid, leaning against the orthostats at the rear. The corridor walls were an integral part of this circular crown and were erected at the same time. The pavement of the corridor seems to have been laid once the lower course of the crown was installed, since the small slabs that make it up rest against the blocks that serve as walls of the corridor and against the orthostats that form the entrance pair. These small slabs also bear traces of shaping and seem to have been adjusted to the morphology of the slabs that make up the corridor walls.

The roofing system began with the first row of corbels, placed above the orthostats and on the crown foundation course. Once this first row was in place, the second and third courses of the crown were installed. The second row of corbels followed, resting on the first level of the corbelled roof and on the crown upper courses. The corbelled roof and the crown seem to have been put in place simultaneously, alternating between the courses of the crown wall and the rows of corbels until the complete formation of the roof. Indeed, the corbels of the first row rest on the lower course of the crown, while the remaining corbel of the second row rests on the second course of the crown. The roof of chamber ST_118 has largely disappeared today, but some of the blocks identified outside the tomb space could be interpreted as former corbels due to the distinctive shape of blocks used for the corbelled roofing of chambers.

#### Third Level: The Study of Monoliths

**Fig. 14:**
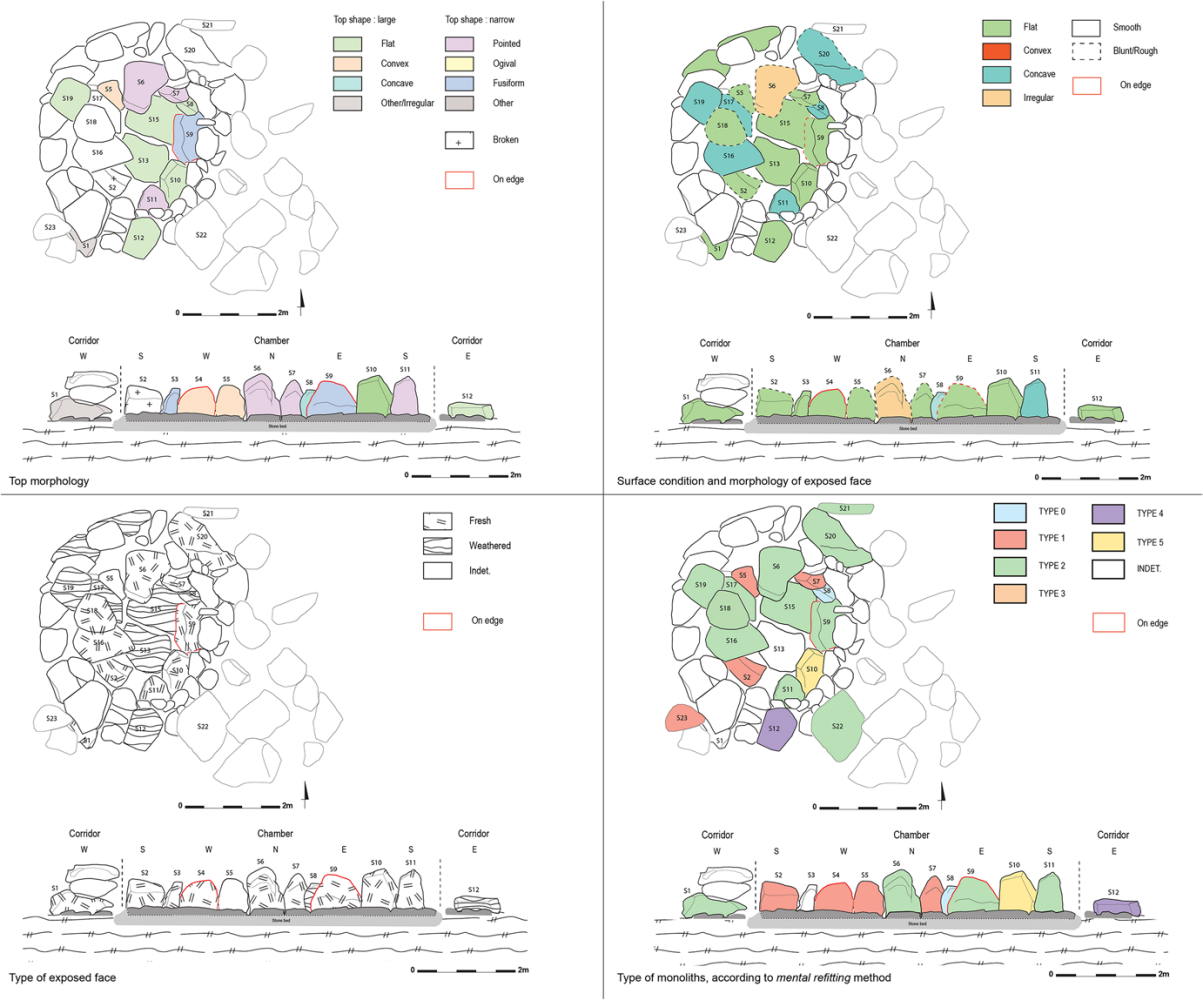
Criterion applied on ST_118’s monoliths (top left: top morphology; top right: surface condition and morphology of the exposed face; bottom left: type of the exposed face; bottom right: type of monoliths according to mental refitting method)

**Fig. 15:**
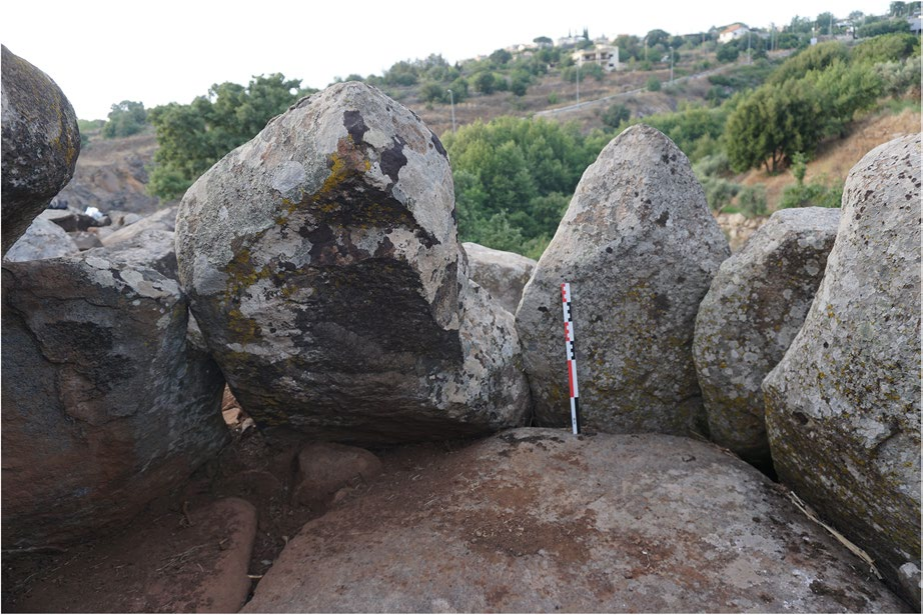
Orthostats facing the corridor - ST_118

**Fig. 16:**
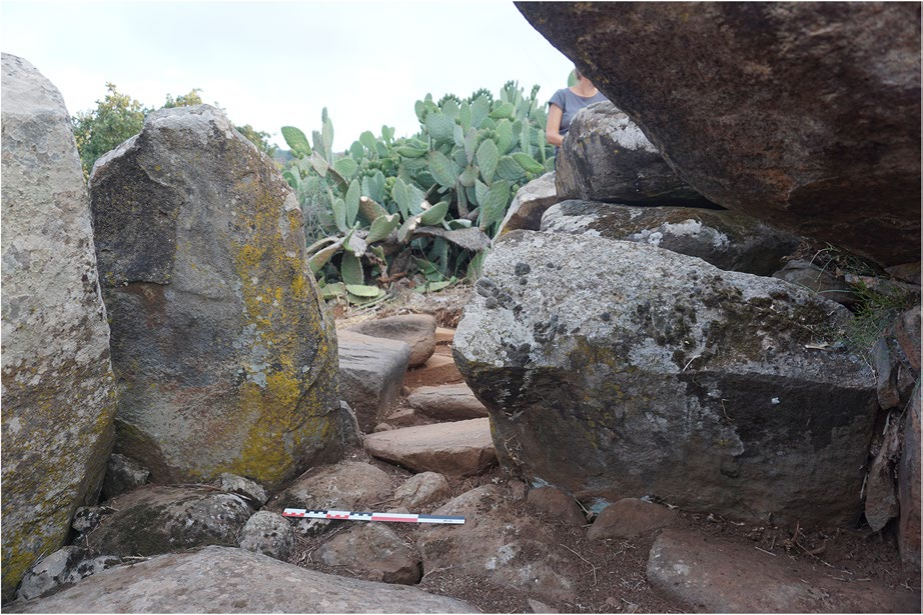
The entrance orthostat pair of ST_118

The good preservation of the chamber allows us to observe the composition of the walls and the intended dynamics during the placement of the blocks (Fig. 14). Mirror effects and singularities are thus visible in the layout of the chamber’s orthostats. First, the middle section of the chamber’s eastern and western walls each feature an orthostat positioned on an edge and facing each other. These are the only orthostats placed in this way in the chamber. The two orthostats making up the entrance pair, located on the southern wall of the chamber, share many similarities. Even though the western block is broken, and half of its height has now disappeared, it must have had the same dimensions as the eastern block, as evidenced by their respective thicknesses, 0.26 m for the western orthostat and 0.27 m for the eastern one. Regarding the northern wall, the orthostat facing the corridor is much larger than the other orthostats in the chamber. We also observed material removal on its eastern edge, giving it a slight shoulder and consequently a distinctive anthropomorphic form. The orthostat directly to the east of this block, also facing the entrance, presents a similarly distinctive shape, also qualified as anthropomorphic. These two blocks thus stand out from the other orthostats, and their specific position facing the corridor suggests that their placement within the chamber was a deliberate choice. The observation of the shape of the tops of the monoliths provides valuable information about the symbolic dynamics sought by the builders of these megalithic tombs. Indeed, we noted that the orthostats with a narrow and pointed top were placed in specific locations within the chamber: facing the corridor and at the entrance of the chamber (Figs. 15 and 16).

Concerning the general shape and surface condition of the exposed face of the chamber blocks, many orthostats have a flat surface, except for the eastern orthostat of the entrance pair, which presents a concave face, and the orthostat facing the entrance, whose exposed face is irregular. Regarding the entrance orthostat pair, the exposed face of the western block has been defined as flat. It is however broken, and therefore it is difficult to determine its original shape with certainty and thus any potential symmetry with the eastern block according to this criterion. The three blocks of the pavement have a flat and smooth surface, providing a perfectly level floor.

Regarding the corbels, the blocks of the first row present a concave lower surface, corresponding to the exposed face and a flat face on the upper side. The blocks in the second row, on the other hand, have a flat face on the lower side. This arrangement of the blocks ensures the roof’s stability, since the concave face of the corbels in the first row fits perfectly on top of the orthostats on which they rest, and the flat face accommodates the second row of corbels.

The slabs that make up the corridor walls have a flat face on the exposed side, *i.e*., facing the corridor. This creates an impression of regularity and straight walls.

Observing the shape of the exposed face of the blocks in the crown tells us that their arrangement is far from random. Indeed, most of these blocks have a flat face on their exposed side, *i.e*., their outer face. Once again, this gives an impression of regularity and ensures the circular shape of the crown. This crown is particularly well-built, as the massive blocks constituting the foundation have a flat upper face, ensuring overall stability and facilitating the placement of the upper layers.

Identifying the type of exposed face according to D. Sellier’s method, *i.e*., the distinction between the weathered face and the fresh face, provides insights into the choices made by the builders in arranging the blocks. Through the example of ST_118, it appears that a specific pattern has been established within the chamber. Indeed, the orthostats almost systematically present a fresh face as the exposed face, while the pavement’s blocks have a weathered face on the exposed side.

Regarding the distribution of monolith types identified using E. Mens’ mental refitting method, we can observe that most of the employed blocks come from the surface levels of the basalt outcrops and correspond to types 1 and 2. Two monoliths, however, seem to come from the deeper levels of the original outcrop – the orthostat forming the southeastern corner of the chamber, which is of type 5, and the lower slab of the eastern wall of the corridor, of type 4. Facing the type 5 orthostat, another orthostat positioned at the southwestern corner of the chamber, whose type could not be determined in the field due to significant erosion of the block, presents many angular faces and may correspond to type 4 or 5. Another mirroring relationship can be considered between these two orthostats.

All the blocks that make up the megalithic tomb ST_118 are formed of non-vesicular basalt (B0). The supply of basalt seems to come from a unique extraction zone.

### Tomb ST_129

**Fig. 17:**
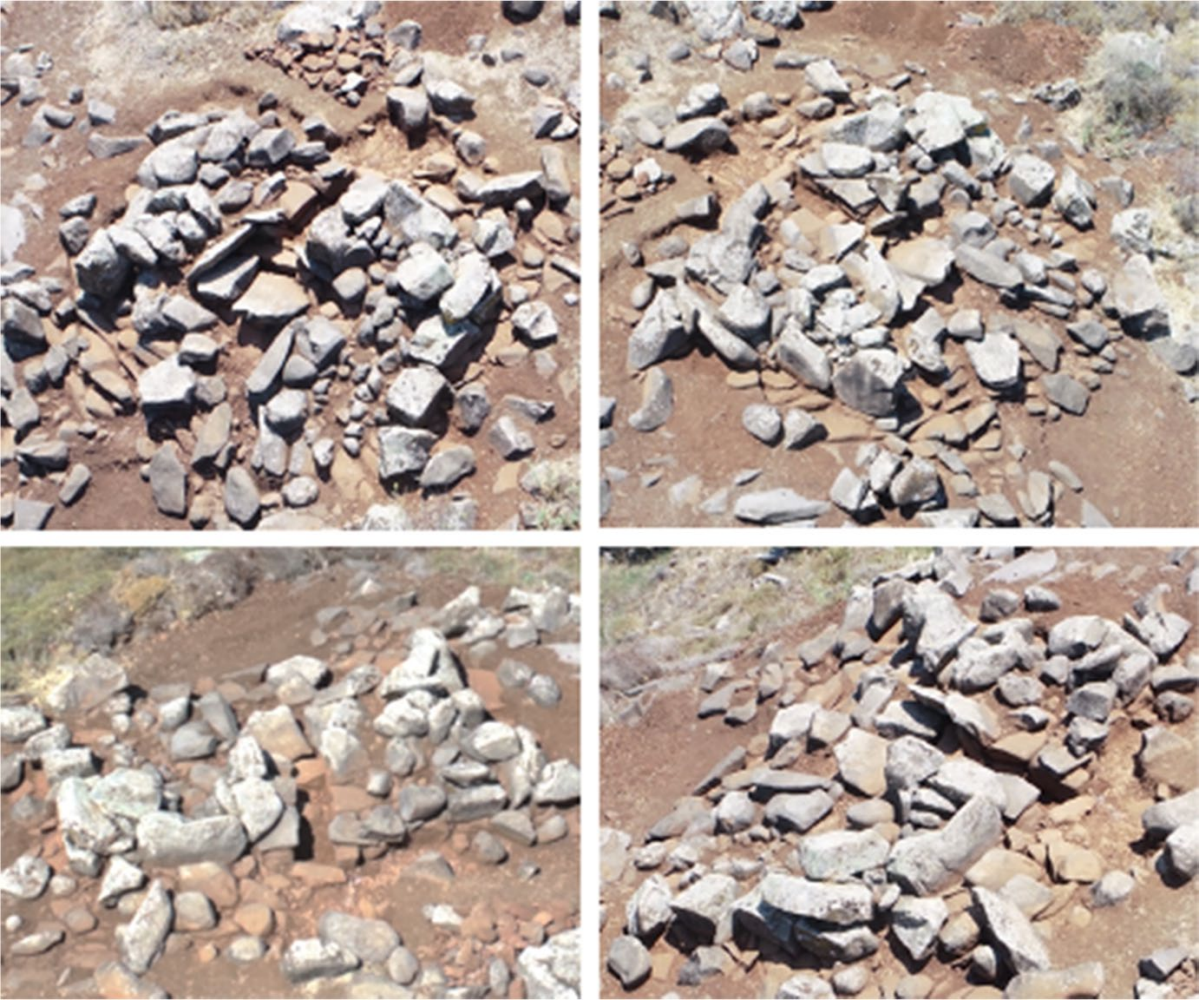
UAV views of megalithic monument ST_129 (top left: from the north; top right: from the east; bottom left: from the south; bottom right: from the south-west) (© DGA). The preserved internal diameter of the chamber is of 2.30 m from northeast to southwest; the preserved external diameter of the crown is of 4.30 m from east to west

Megalithic tomb ST_129 was studied and excavated during the first campaign of the MEG-A project in the summer of 2022 (Fig. 17). It was mentioned during the first investigations by M. Tallon in 1959, but was not emptied by the workers, as we found it in the same state of preservation as shown in the photograph accompanying his notebook.

Tomb ST_129 is located in the lower part of the Kroum Metowmeh cemetery, on the slope leading down to the Wadi Moungez to the north. Like ST_118, which also lies on this slope, ST_129 was erected on a terrace. The terrace wall, however, which was particularly well-preserved over about 40 m, has been washed away in this part of the terrain, contributing to the collapse of ST_129. Even though it shares this same terrace wall, ST_129 is located at a significant distance from other monuments, separated by a wall perpendicular to the slope (ST_130) (Fig. 8).

#### First Level: Plan and Volume

**Fig. 18:**
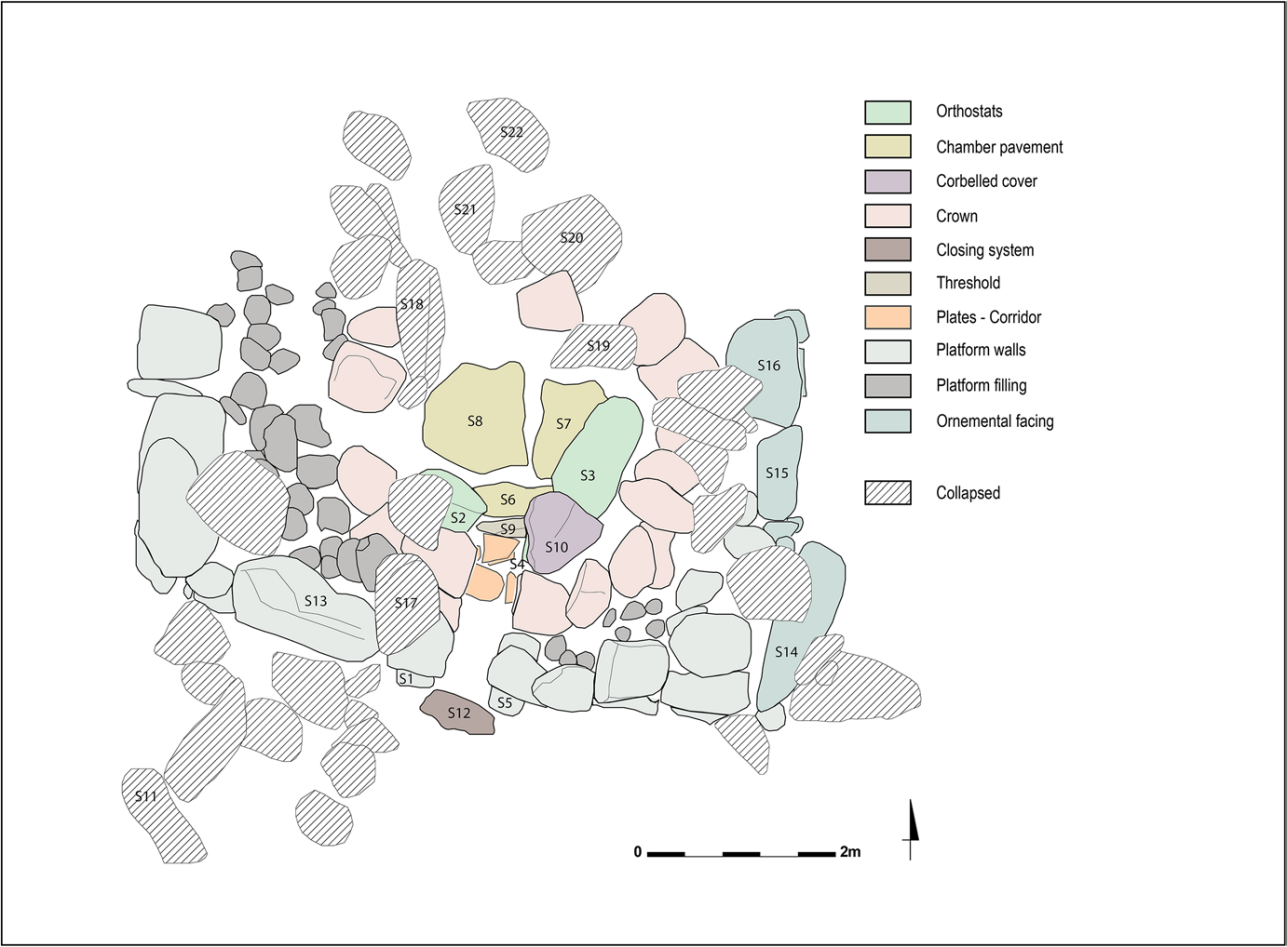
Spatial distribution of ST_129 components (link to 3D model - Sketchfab: https://skfb.ly/oTrzD)

The northern half of ST_129 has slid down the steep slope in this area (Fig. 18). This megalithic tomb consists of a circular chamber with only three orthostats still in place, positioned to the south and east. The chamber was originally delimited by several other orthostats that have now slid down. The chamber’s pavement consists of three massive blocks, but like the orthostats, other blocks would have been used, especially to the north. This pavement rests on a stone bed arranged on the substrate, aiming to level and stabilize the ground. It is challenging to determine the precise boundaries of ST_129’s chamber due to its collapse to the north. It is therefore difficult to determine the exact limits of the chamber. As with ST_118, the orthostats of ST_129 are held at the front by the pavement and at the rear by the crown, rather than being embedded in a pit dug into the ground.

The chamber, which appears to follow a circular plan with a preserved diameter of 2.30 m (northeast/southwest), is part of a circular crown measuring 4.30 m in diameter from east to west. This crown serves to support the rear of the three preserved orthostats. The arrangement of blocks in this crown is the only clue we have to propose a restitution of the chamber’s limits, as well as its circular shape. The crown is made up of medium and large-sized blocks, arranged in a facing of which only the lower course is preserved. The crown has been severely disrupted by the collapse of the structure to the north, making its limits challenging to define precisely.

**Fig. 19:**
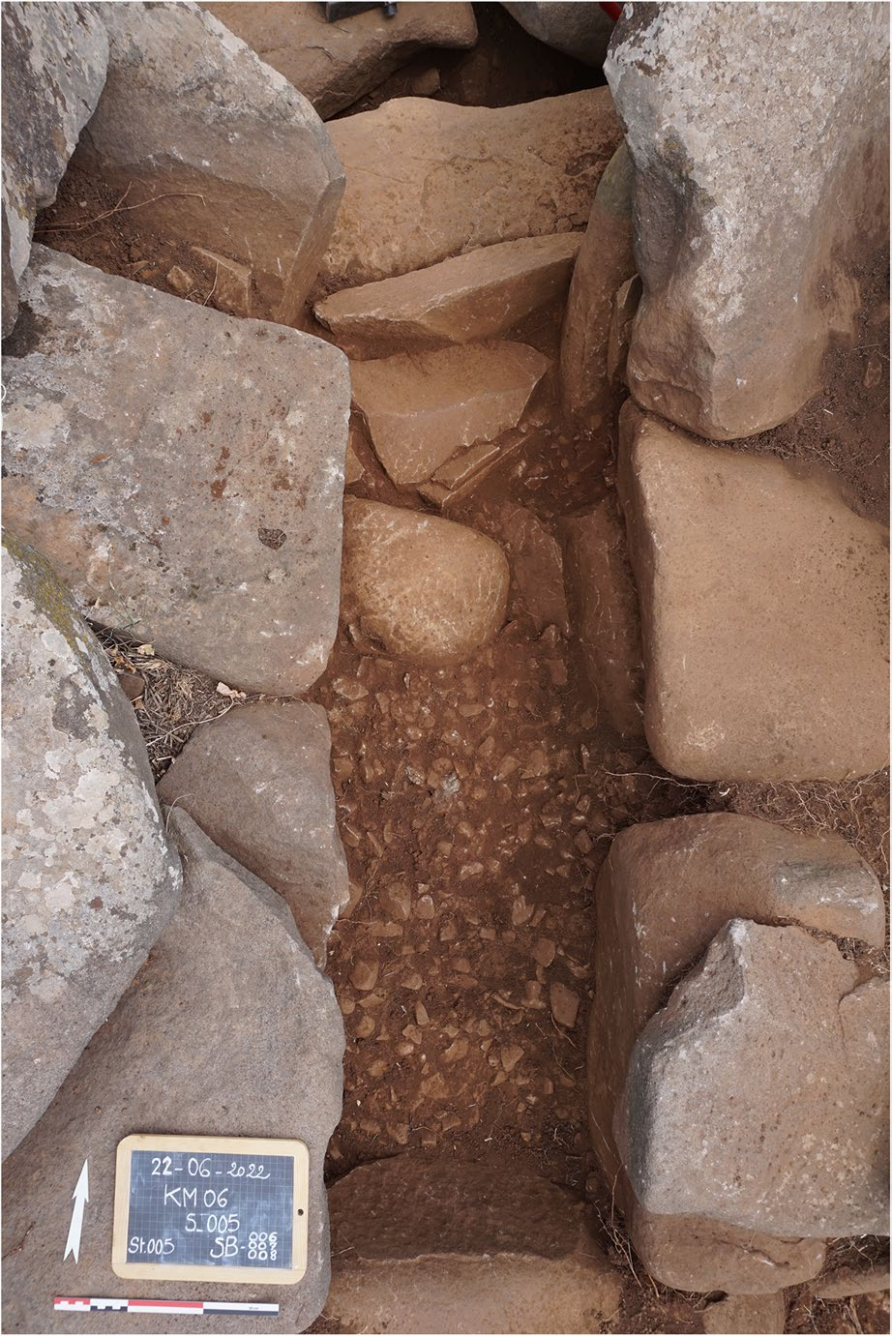
Corridor of ST_129 after excavation, from the south

The chamber is accessible through a corridor oriented south-southwest, measuring 1.75 m in length and 0.56 m in width, with well-preserved east and west walls (Fig. 19). These walls were constructed in two distinct parts and are integrated into two different building units. The northern half of the walls is part of the circular crown, while the southern half is part of the quadrangular platform. The remains of both units were built in two courses, with a large slab constituting the lower course and a smaller slab as the upper course. For the southern half, the lower course supports the corner chaining with the southwestern and southeastern walls of the platform.

**Fig. 20:**
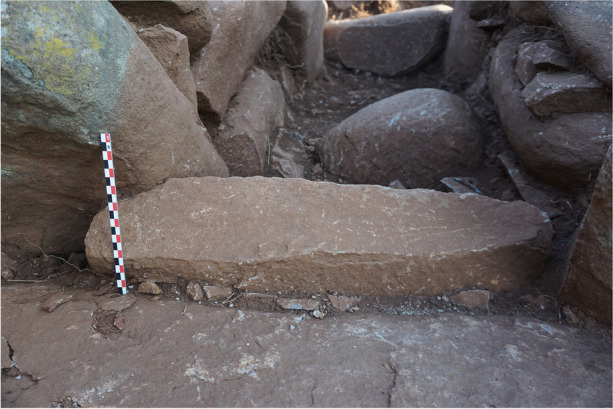
Threshold slab to the north of ST_129’s corridor, from the north

**Fig. 21:**
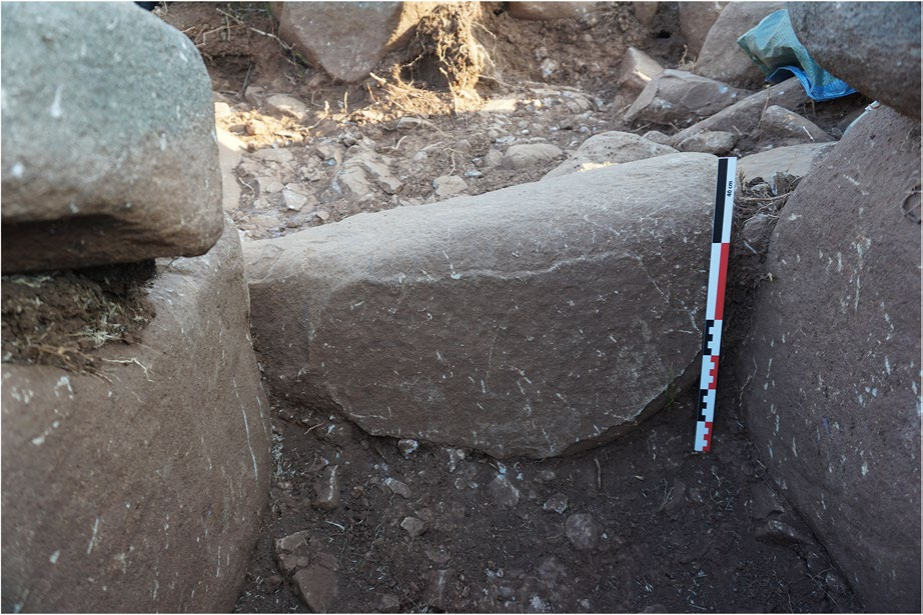
Closing block at the southern end of ST_129’s corridor, from the north

At the northern end of the corridor, a slab on edge stands between the two entrance orthostats, marking the transition zone between the corridor and the chamber (Fig. 20). This threshold slab serves as a demarcation point. At the southern end of the corridor, a block has been placed probably to close this space (Fig. 21).

Excavation revealed the circulation level of the corridor, consisting of a thick layer of well-packed gravel that extends along its entire length. No paving was discovered during the excavation. However, a specific floor arrangement was uncovered north of the corridor. The space just before entering the chamber, near the threshold slab, is marked by several plaques, forming a clear delineation between the corridor and the chamber.

A quadrangular platform envelops the chamber and the crown, delineating the space of the tomb. This platform was built using large module blocks, forming a single-face dry stone wall. The different walls of the platform are not identical. We can distinguish the southwestern and western walls, whose masonry is composed of massive blocks, which we could describe as Cyclopean, and the southeastern and eastern walls, where the blocks are smaller and exhibit a more regular module. Cleaning the exterior of the tomb revealed the remains of the collapse of the southwestern wall of the platform. Several fallen blocks that formed the upper courses of this portion of the platform were uncovered in front of this wall. In view of their layout, these blocks must originally have been stacked on top of each other and likely constituted the three or four upper courses above the blocks still in place. This suggests that the platform of tomb ST_129 must, at some point in its history, have had considerable dimensions and likely concealed a significant portion of the crown and the chamber.

In front of the platform’s eastern wall, an arrangement of three upright slabs has been set up. This alignment serves as the external facing of the eastern wall of the platform and appears to have served an ornamental function. Indeed, it stands out in terms of its appearance and the dimensions of its blocks when compared to the walls of the platform. The northern wall, on the other hand, has completely disappeared, sliding down the slope.

The space between the external walls of the platform and the crown has been filled with small-module blocks. This filling appears more substantial to the west, giving the impression that the chamber is slightly offset to the east compared to the median axis of the structure.

#### Second Level: Operating Chains and Architectural Phasing

**Fig. 22:**
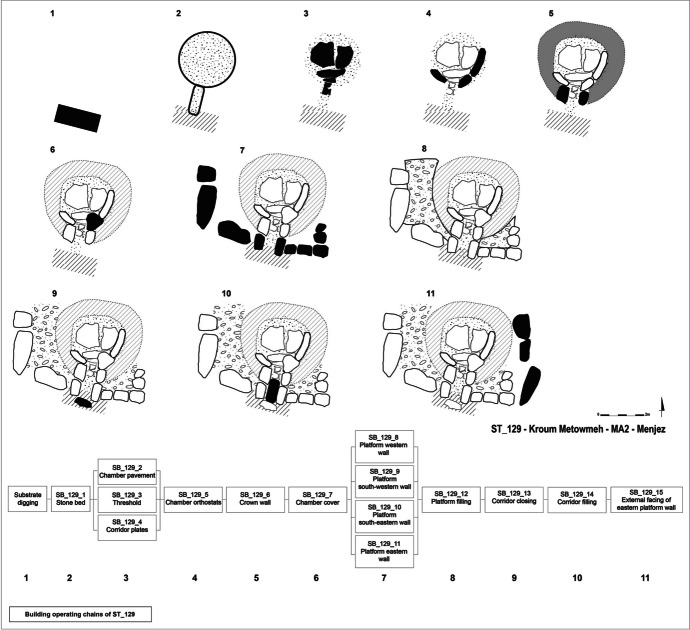
Building sequence with stratigraphic building units of ST_129

Considering the state of preservation of the megalithic tomb ST_129, we must note that a portion of the building sequence of this monument has now vanished. From the remaining elements, the building sequence of ST_129 is divided into eleven stages, from site planification to the completion of the structure (Fig. 22).

First, before the erection of the structure, the terrain was prepared. The exposed substrate was partially excavated, particularly in the southern part of the tomb, and parts of the platform were then set in. This manoeuvre resulted in a leveled terrain before the installation of the tomb.

Installation of the chamber begun with the placing of a stone bed on the substrate surface, leveling the space to stabilize the entire chamber. This stone bed was placed in the chamber’s space, but also in the area of the future corridor, since the plates uncovered during the excavation rest on this stone bed level. The chamber’s pavement, composed of large blocks, was then placed above. To enhance the stability of this pavement, several wedging stones were positioned between the paving blocks and the stone bed. Filling stones were then inserted into the gaps between the paving blocks to eliminate any voids. The threshold slab, marking the transition between the corridor and the chamber, was subsequently placed to the south of the entrance paving block, as well as the plates to the north of the corridor.

Once the pavement had been installed, the orthostats forming the chamber’s walls were set up. These were positioned against the pavement, sometimes with intermediate wedging stones. As such, the chamber’s pavement serves to support the orthostats at the front. The circular crown wall supports the orthostats at the rear. The northern part of the corridor walls was erected at this point. These, along with the other blocks of the circular crown, support the entrance orthostats of the chamber. Finally, the corbelled roof of the chamber was placed. It rested on the chamber’s orthostats and the circular crown wall.

The builders then erected the quadrangular platform within which the chamber and the circular crown are found. The blocks of the external walls of this platform were stacked on top of each other. Two blocks of the southern wall of the platform form the southern half of the corridor walls. The lower slabs support the corner chaining with the second course. Once the corridor walls were completed, and the walls of the quadrangular platform set up, the space separating them from the crown was filled with small stones. It is then conceivable that the corridor’s closing block was placed south of the corridor. This stage was followed by the filling of the corridor with a thick layer of gravel running all along the length of the corridor.

An alignment of massive blocks, probably constituting an ornamental facing, was then placed in front of the eastern wall of the quadrangular platform. This alignment rested directly on the substrate, sometimes with a few intermediate wedging stones to stabilize the blocks. It may be a later addition, although there is no evidence to support this hypothesis. This alignment almost certainly constitutes the last addition. Besides its ornamental purpose, the installation of this facing seems to aim at restoring the symmetry of the overall structure. Thus, before the installation of this facing, the chamber was likely slightly offset to the east compared to the median axis.

As for the architectural phasing of megalithic tomb ST_129, we consider that it was probably built in a single phase and at a single moment, dealing with a single architectural project. This hypothesis is supported by several concordant elements. Firstly, the basalt substrate was excavated and leveled in a first building stage in the southern part of the monument, at the level of the southern walls of the future quadrangular platform. Secondly, the stone bed, laid during the second building stage, was not only positioned within the space of the future chamber but also extended along the entire length of the future corridor. These first two elements indicate that the various architectural spaces that constitute ST_129 were anticipated and planned in advance by the builders, from the early stages. This seems to invalidate the hypothesis of two distinct phases, considering a former one with a round cairn, *i.e*., the chamber and the circular crown, and a short corridor, and a latter one with an extended quadrangular platform and corridor.

The megalithic monument ST_117^8^, studied and modeled in 3D in 2018^9^, also located in the Kroum Metowmeh sector, presents the same characteristics as ST_129 and seems to belong to the same type. For this monument, we can say with certainty that it results from a single phase and a single architectural project. The walls of its corridor are preserved on two courses, with a lower level composed of a large slab running almost the entire length of the corridor and an upper level consisting of two smaller blocks (Steimer-Herbet *et al*., 2019a, b, p.216). Like ST_129, these two blocks each fit into two distinct spaces, the northern block fits into the circular crown surrounding the chamber, while the southern block fits into the sub-rectangular enclosure surrounding the round cairn. The fact that the lower level supports these two blocks and is made up of a single large block leaves no doubt about the architectural phasing of ST_117. By proceeding analogously, we can then support the hypothesis of a single phase also for ST_129.

##### Third Level: The Study of Monoliths

**Fig. 23:**
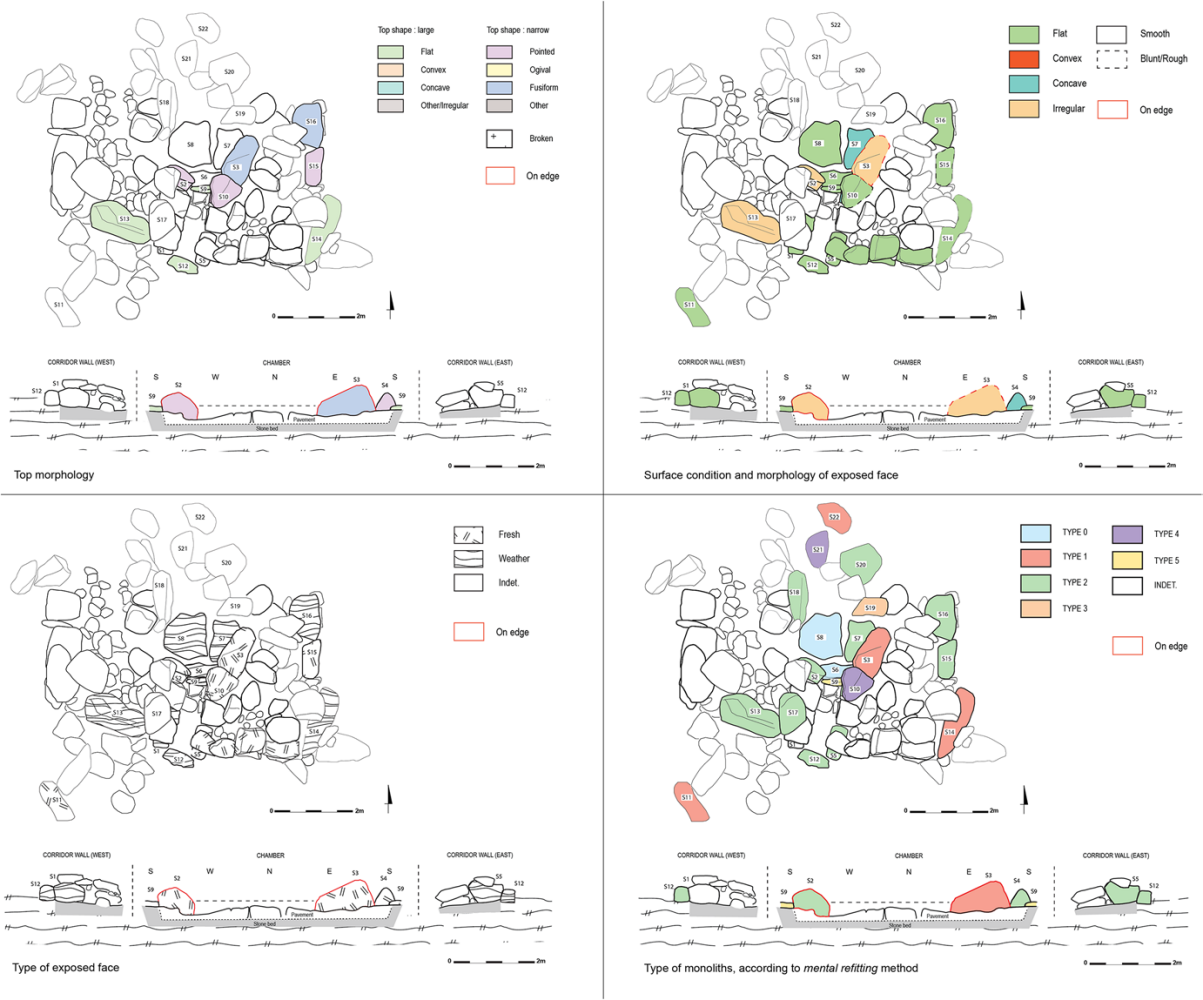
Criterion applied on ST_129’s monoliths (top left: top morphology; top right: surface condition and morphology of the exposed face; bottom left: type of the exposed face; bottom right: type of monoliths according to mental refitting method)

Due to the collapse of the northern half of tomb ST_129, and unlike tomb ST_118, it is challenging to grasp the full dynamics sought by the builders in the installation of the various monoliths in the chamber. We have limited evidence, with only three orthostats still in place today. Nevertheless, some interesting elements emerge from the study of the monoliths in tomb ST_129 (Fig. 23). As seen in the case of ST_118, there are indications of a symmetrical relationship between the two orthostats forming the entrance pair. These are of equivalent height, with the western orthostat measuring 0.79 m and the eastern orthostat measuring 0.73 m. Additionally, both blocks have a narrow and pointed top, similar to what was observed on the eastern orthostat of the entrance pair in ST_118. However, differences between these two orthostats have also been noted—the western orthostat is arranged on an edge, while the eastern orthostat is arranged vertically. Furthermore, the western orthostat is significantly wider, measuring 0.90 m, while the eastern orthostat is 0.44 m wide. The other remaining orthostat that forms the eastern wall of the chamber has been arranged on an edge.

The only remaining corbel of the corbelled roof of the ST_129 chamber has a narrow and pointed top. This type of summit has also been observed on the orthostats of the entrance pair, creating a mirroring effect between these two blocks that mark the entrance to the chamber. The third orthostat has a summit defined as fusiform. Most other blocks in tomb ST_129 have a wide and flat top, ensuring a solid base for the stacking of other blocks, especially concerning the walls of the platform.

Regarding the criterion of the shape of the exposed face of the monoliths in tomb ST_129 and their surface condition, certain points are worth noting. The monoliths positioned in the corridor (*i.e*., the entrance slabs that make up the walls, the threshold slab, and the closing block) all have a smooth, flat face on their exposed side. This creates an effect of regularity in the corridor space, giving it the shape of a perfect rectangle, with rectilinear contours.

This rectilinear and regular aspect produced by the exposure of the flat faces of the blocks was also observed in the platform space, notably for its western, south-eastern, and eastern walls. This reinforces the platform’s perfectly quadrangular appearance.

With regard to the ST_129 chamber, we note that the eastern orthostat of the entrance pair has a smooth, concave face on the exposed side, which rests on the threshold slab. Its concave shape makes it easier to adapt to the morphology of this threshold slab. The western orthostat of the entrance pair also has a smooth but irregular exposed face. This orthostat does not rest against the threshold slab, which is positioned behind the orthostat towards the corridor. For this criterion, no play of symmetry is identified between the two orthostats forming the entrance pair. As far as the corbelled roof of the chamber is concerned, only one corbel is still in place. Its exposed face (*i.e*., its lower side) is flat. This configuration necessitates the installation of several wedging stones between the corbel and the back of the orthostat on which it rests. The upper face of the corbel is also flat, making it easier to install the second row of corbels.

The three remaining orthostats in the chamber present a fresh face on the exposed side, while the three paving blocks have a weathered face.

Concerning the platform, the exposed faces of the blocks on the south-eastern wall, *i.e*., those facing the outside of the structure, are of the fresh type. Fresh faces are generally more regular and flatter than weathered faces, which have been exposed to erosion and therefore show irregularities (Mens, 2008, p.26). The positioning of the flat fresh faces of the blocks in this platform wall is a deliberate choice, aimed at creating a regular, straight-looking wall.

As for the typology of the blocks used, according to the *mental refitting* method, we were able to determine that a large proportion of the monoliths studied correspond to types 1 and 2 and therefore come from the upper levels of the original outcrop. We can also see that two of the blocks making up the paving are type 0, *i.e*., isolated blocks, which were selected by the builders probably due to their suitability for this function. We have also identified three monoliths corresponding to types 4 and 5, extracted from the lower levels of the original outcrop. Interestingly, two of these monoliths occupy a very specific function within the structure. One corresponds to the remaining corbel of the chamber’s corbelled roof, whose function requires a specific shape, and the other to the threshold slab, which marks the transition zone between the corridor and the chamber. The third block, identified as type 4, is no longer in place and has slid down the slope to the north. In view of its unusual shape, it has been identified as a potential orthostat or corbel. By comparison with the remaining corbel, also of type 4, we suggest that it is a former corbel.

Of the monoliths studied for tomb ST_129, only one is made of slightly vesicular basalt (B1), corresponding to one of the corridor’s cover slabs, moved outside the structure during excavation. The rest of the blocks are made of non-vesicular basalt (B0).

The particularity of this megalithic tomb lies in the placement of a purely ornamental facing in front of the platform to the east of the structure. The arrangement of these three upright monoliths does not appear to respond to any technical need, since they are three upright monoliths, one with a narrow, pointed top and another with a fusiform top, invalidating the idea that this alignment was included in a dry-stone wall (Laporte *et al*., 2017). Observation of the morphology and type of exposed face of these three blocks supports the idea that this is a purely ornamental facing. Each of these three upright slabs has a flat exposed face. The southern block has now slipped to the east, but this alignment was originally intended to form a perfectly regular and rectilinear facing. The composition of the facing also follows a rhythm illustrated by the alternation between weathered and fresh faces. These different observations seem to confirm that the application of our methodology to the study of the monoliths of this megalithic tomb allows us to confirm the ornamental and potentially symbolic character of this facing.

## Discussion

Applying our unique Levantine megalithic building techniques methodology through these two megalithic tombs in the Kroum Metowmeh sector illustrates the wealth of information we can extract when mobilizing such methods.

At the first reading level, we identify two tombs that seem to belong to two different types. ST_118 corresponds to a tomb comprising a circular chamber associated with a circular crown, accessible through a paved corridor. ST_129, on the other hand, corresponds to a tomb with a chamber that is probably circular in plan, associated with a circular crown, accessible through an unpaved corridor, and a quadrangular platform. The major difference lies in the presence or absence of a platform surrounding the chamber and crown area. However, there are many similarities. A circular shape of the chamber and crown has been observed in both tombs. In both cases, the chamber is covered by a corbelled roof and accessible through a corridor facing south-southwest. The south-facing orientation of the tomb seems to be a constant in the megalithic architecture of Menjez and has been systematically observed in the other megalithic tombs studied.

Regarding the second level of reading, which consists of reconstructing the operative chains that condition the building of these megalithic monuments, we can first point out that tomb ST_129 is the result of a more complex construction process than ST_118. Indeed, ST_129 was built through eleven stages of construction, which, after the chamber and crown space, include the installation of a platform and an ornamental facing. However, the same construction process was observed for ST_118 and ST_129. In fact, the installation of the chamber and crown space follows the same pattern for both monuments presented. In both cases, the ground is prepared with the installation of a stone bed, after the outcropping substrate has been dug in the case of ST_129. This is followed by the pavement, the orthostats, the crown, and the corbelled cover. As a result, the same workflow is used for both types of tombs. Moreover, tombs ST_118 and ST_129 each seem to correspond to a single architectural project, with no breaks or reworking observed in the architecture. These two monuments would therefore have been erected at the same time. A nuance needs to be added in the case of ST_129, however, for which it is quite conceivable that the outer facing of the platform’s eastern wall was added to the structure at a later stage.

By applying the methodological tools used in buildings archaeology, we were able to piece together the construction workflows of these megalithic monuments and thus determine the various stages of construction.

Regarding the third reading level, it would seem that particular attention has been paid to some monoliths, namely the orthostat facing the entrance for ST_118, as well as the two orthostats making up the entrance pair. Some of the observations made at Menjez point to repetitions between the different monuments, such as the choice of a monolith with a narrow, pointed top for the corbels, as observed on ST_129. The observation of this characteristic shape is a good indicator for spotting ancient corbels among the collapsed blocks around megalithic structures. Some orthostats also feature this type of top, but it is not a constant; they are generally positioned at very specific points in the chamber, as we have seen on ST_118 and on ST_129 and its pair of entrance orthostats. The orthostat on edge, which forms the eastern wall of the chamber, has a fusiform top. We also noticed this in tomb ST_118, where the orthostat forming the median part of the east wall has a fusiform top.

Tomb ST_118 is particularly interesting for the restitution of the symbolic coding expressed in the architecture, due to the perfect preservation of the chamber walls, the space where the games and rhythms revealing this symbolic dimension are most present. If we extend our comments to the whole of our corpus, we have observed that these monoliths in particular stand out recurrently from the others. The application of various analysis criteria has shown that the blocks were deliberately selected for their architectural properties and symbolic potential.

Concerning the geological criteria, ST_118 and ST_129 are composed of non-vesicular basalt (B0). These two monuments are located in the lower part of the Kroum Metowmeh necropolis, and according to field observations, the vesicularity that characterizes Menjez basalt seems to vary with elevation. In the lower part of the necropolis, we observed outcrops of non-vesicular basalt (B0), while in the upper part, the basalt is vesicular (B1-B2). The blocks used to build ST_118 and ST_129 were likely quarried in the immediate vicinity of these monuments, in the lower part of the necropolis.

These initial findings allow us to establish that applying a geo-archaeological approach to the study of monoliths provides us with significant information on the symbolic dimension expressed in this megalithic architecture and on the mechanisms for sourcing basalt.

## Conclusion

In this article, we present in detail a unique megalithic building techniques methodology applied for the first time to megalithic monuments in the Levant and that we were able to adapt to the context of the site under study. Using two examples, we demonstrate the validity of our method and the significant results that can be drawn from it. In future research, our methodology will be applied to the entire corpus comprised of the 45 monuments identified in the municipality of Menjez. It will enable us to propose a comprehensive technological study using tools that are unprecedented in Levantine megalithic architecture.

Understanding the supply of basalt is an essential part of our fieldwork. Our interest in the geology and geomorphology of the blocks enables us to reconstitute the pre-Megalithic landscape and understand the supply mechanisms of these megalith-building communities. Geological surveys are currently underway, aiming to develop a geological map of the Menjez region. Based on our initial observations, we have been able to establish that sourcing was a reasoned and opportunistic geological choice. Indeed, the builders erected their monuments in the immediate vicinity of exploitable basalt outcrops, enabling them to obtain easily usable and transportable blocks. The difficulty lay in placing the blocks, some of which were of considerable size and required a great deal of manoeuvring. Building a megalithic monument requires collective effort and cooperation within a community. This raises the question of how to organize such a construction site. The Menjez megalithic tombs do not appear to have been assembled from a single outcrop. Our research suggests that most of the monoliths used in the construction of the monuments came from the upper levels of the outcrops. Blocks from the deeper levels fulfill very specific functions within the structure. This tells us a great deal about the practicality and architectural properties of the monoliths sought by the builders.

The interpretation of symbolic coding, particularly through the observation of geomorphological characteristics of the monoliths used in the construction of megalithic tombs, also represents a significant part of the work carried out in this study. Our observations on the two examples presented in this article show that these communities integrated symbolic elements into the layout of the various monoliths within the monuments. The proposed geoarchaeological approach provides us with strong clues to understand the symbolic coding expressed in this architecture, highlighting the different patterns of texture and rhythms observable in the arrangement of the blocks. We are then able to reveal a facet of the symbolic and cultural identity of these megalithic builder communities. As pointed out by G. Robin, it is essential to remember that these are structures conceived by humans and therefore humanized. It is necessary to conceive this space in its plurality and evoke its multiple nature (Robin, 2016). Indeed, this space should not only be considered as simple architecture, but as the result of a mental construction, reflecting social and symbolic practices specific to these communities. It is therefore necessary to be interested in both the constructed and non-constructed space associated with these megalithic monuments. The application of this methodology is particularly interesting to understand how these monuments, especially their internal space, were organized and perceived (Mens *et al*., 2022, p.7).

This groundbreaking analysis of Menjez’s unique building techniques in Akkar, therefore, has a dual interest and is part of one of the major objectives of the MEG-A project, namely defining the identity of these megalithic communities. It will ultimately allow us to outline the technical and cultural portrait of these megalithic societies in this part of Lebanon in an unprecedented way, through the application of tools previously used only for the study of Western European monumentality. It represents a new way of approaching megalithic architecture in the Near East, where architecture and associated symbolism prove to be more complex than previously envisioned.

## Data Availability

No datasets were generated or analyzed during the current study.

## Footnotes

1 Project CPF-552-17, PI: T. Steimer-Herbet.

2 IKSIO-PAN, Warsaw.

3 Project n°202,350, PI: T. Steimer-Herbet (Laboratoire d’Archéologie Préhistorique et Anthropologie – Université de Genève - Switzerland).

4 Project n°202/39/I/HS3/02993, PI: Z. Wygnańska (Institute of Mediterranean and Oriental Cultures – Polish Academy of Science – Poland).

5 OSL *Optically Stimulated Luminescence* dating.

6 Combination done during the Barnenez Project led by L. Laporte, where both E. Mens (as a researcher) and F. Cousseau (as a PhD student) were involved (Cousseau et Gassien, 2023; Laporte *et al*., 2017).

7 Small (0–30 cm long); medium (30–60 cm long); large (over 60 cm long).

8 The former Dolmen 9 - British Council Project – CPS 552 − 17; P.I. T. Steimer-Herbet (Steimer-Herbet *et al*., 2019a, b).

9 3D model accessible on Sketchfab (F. Cousseau): https://skfb.ly/oTrBq.
